# Supplementary material for: Human Herpes Virus-8 Oral Shedding Heterogeneity Is Due to Varying Rates of Reactivation from Latency and Immune Containment
Source: Viruses. 2025 Nov 13;17(11):1500. doi: 10.3390/v17111500 (PMC12656823; doi:10.3390/v17111500)
Supplement: Supplementary file 1 [file viruses-17-01500-s001.zip › viruses-3906948-supplementary.pdf]

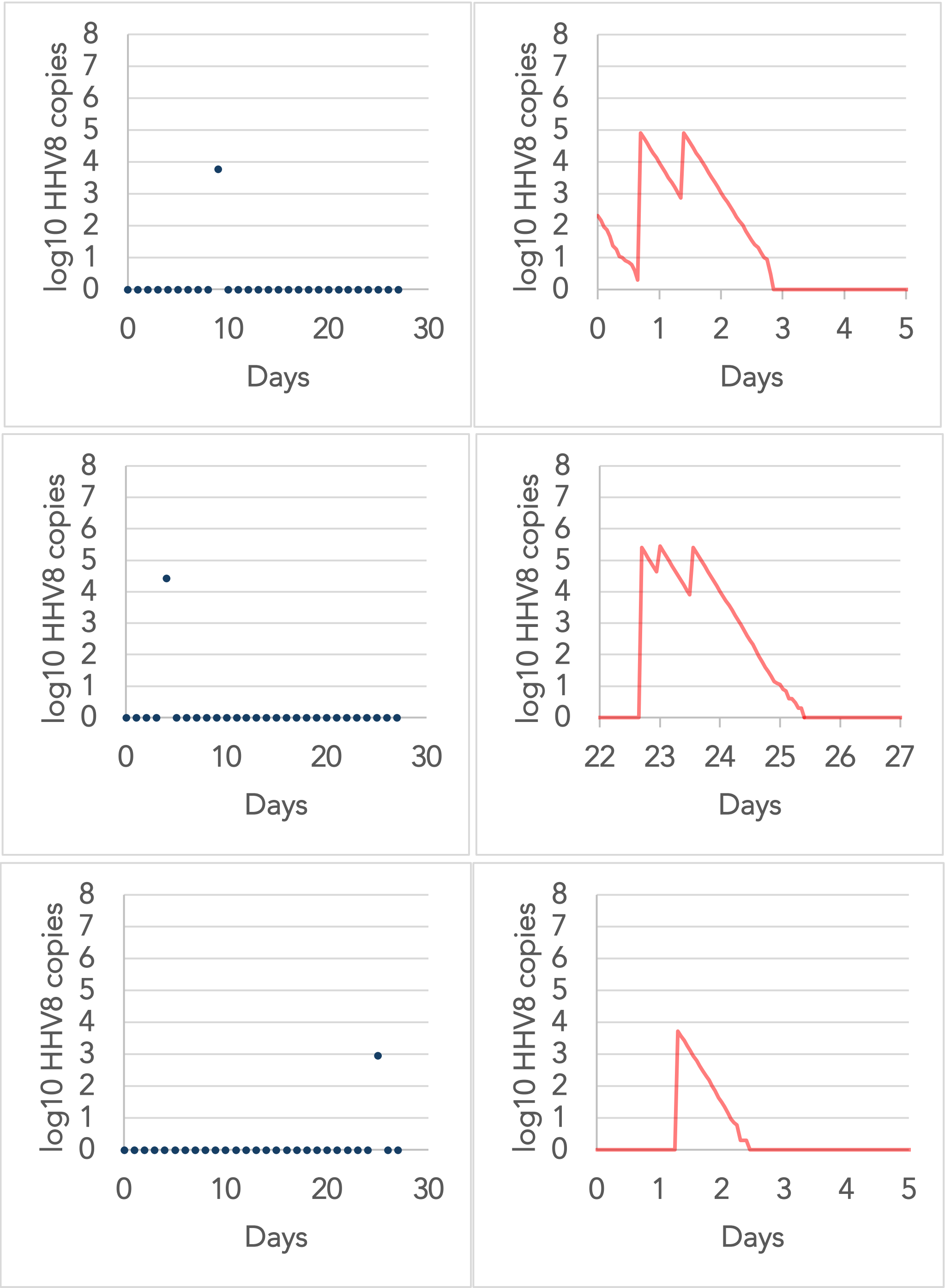
log10 HHV8 copies
8
7
6
5
4
3
2
1
0
0
10
20
30
Days
log10 HHV8 copies
8
7
6
5
4
3
2
1
0
0
1
2
3
4
5
Days
log10 HHV8 copies
8
7
6
5
4
3
2
1
0
0
10
20
30
Days
log10 HHV8 copies
8
7
6
5
4
3
2
1
0
22
23
24
25
26
27
Days
log10 HHV8 copies
8
7
6
5
4
3
2
1
0
0
10
20
30
Days
log10 HHV8 copies
8
7
6
5
4
3
2
1
0
0
1
2
3
4
5
Days

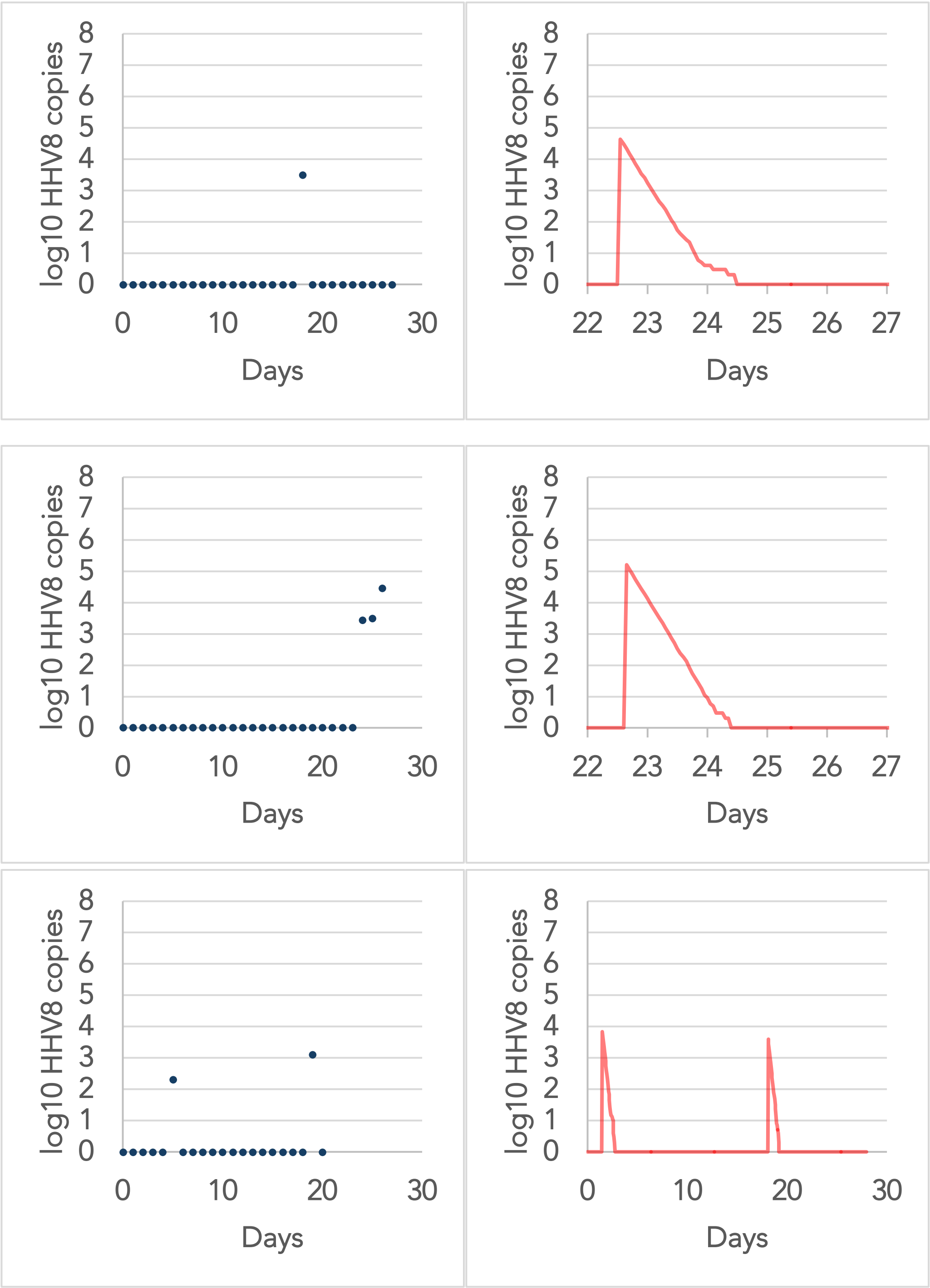

log10 HHV8 copies
0 1 2 3 4 5 6 7 8
0 10 20 30
Days
log10 HHV8 copies
0 1 2 3 4 5 6 7 8
22 23 24 25 26 27
Days
log10 HHV8 copies
0 1 2 3 4 5 6 7 8
0 10 20 30
Days
log10 HHV8 copies
0 1 2 3 4 5 6 7 8
22 23 24 25 26 27
Days
log10 HHV8 copies
0 1 2 3 4 5 6 7 8
0 10 20 30
Days
log10 HHV8 copies
0 1 2 3 4 5 6 7 8
0 10 20 30
Days

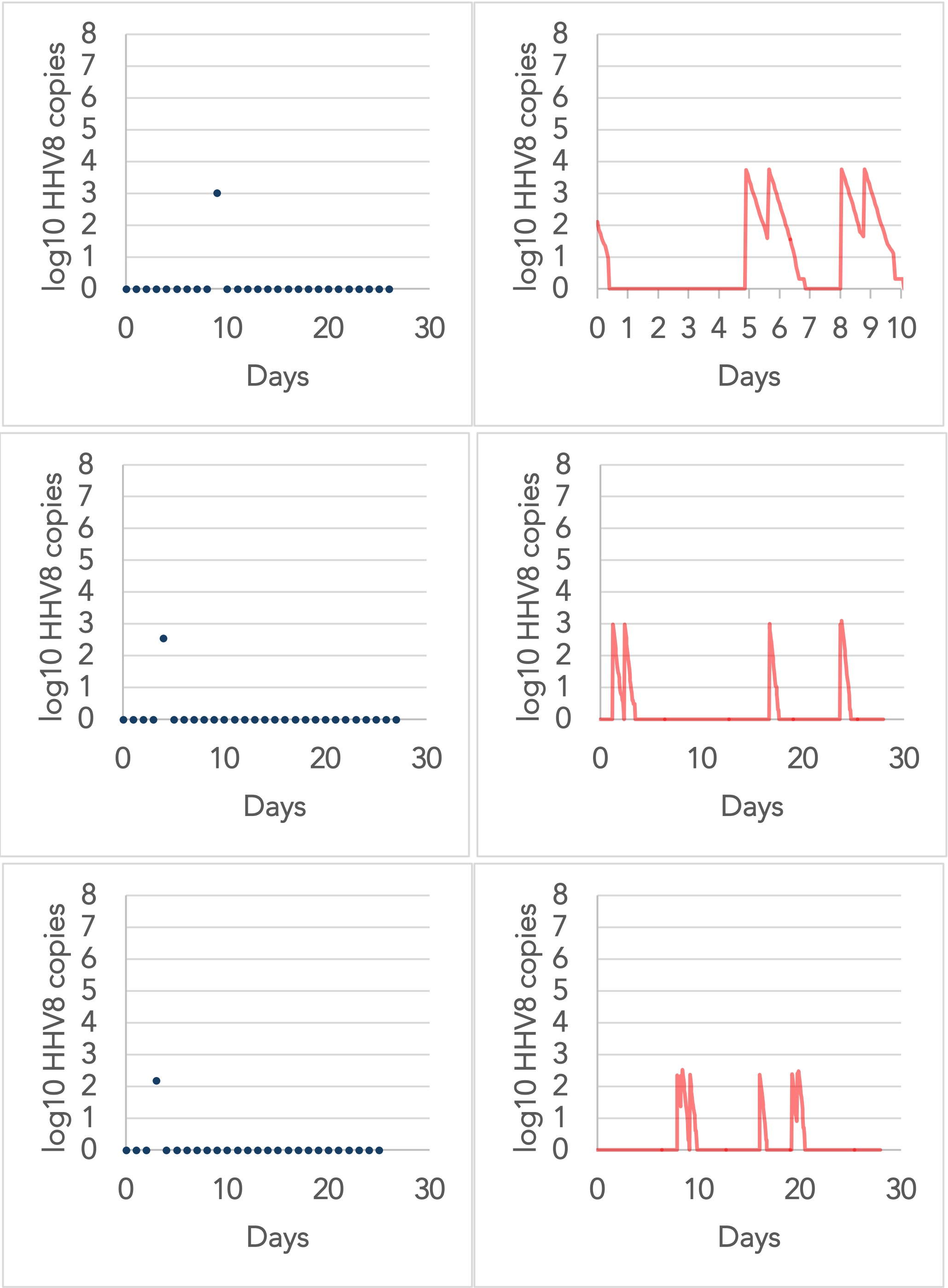

log10 HHV8 copies
0
1
2
3
4
5
6
7
8
Days
0
10
20
30
log10 HHV8 copies
0
1
2
3
4
5
6
7
8
Days
0
1
2
3
4
5
6
7
8
9
10
log10 HHV8 copies
Days
log10 HHV8 copies
Days
log10 HHV8 copies
Days
log10 HHV8 copies
Days

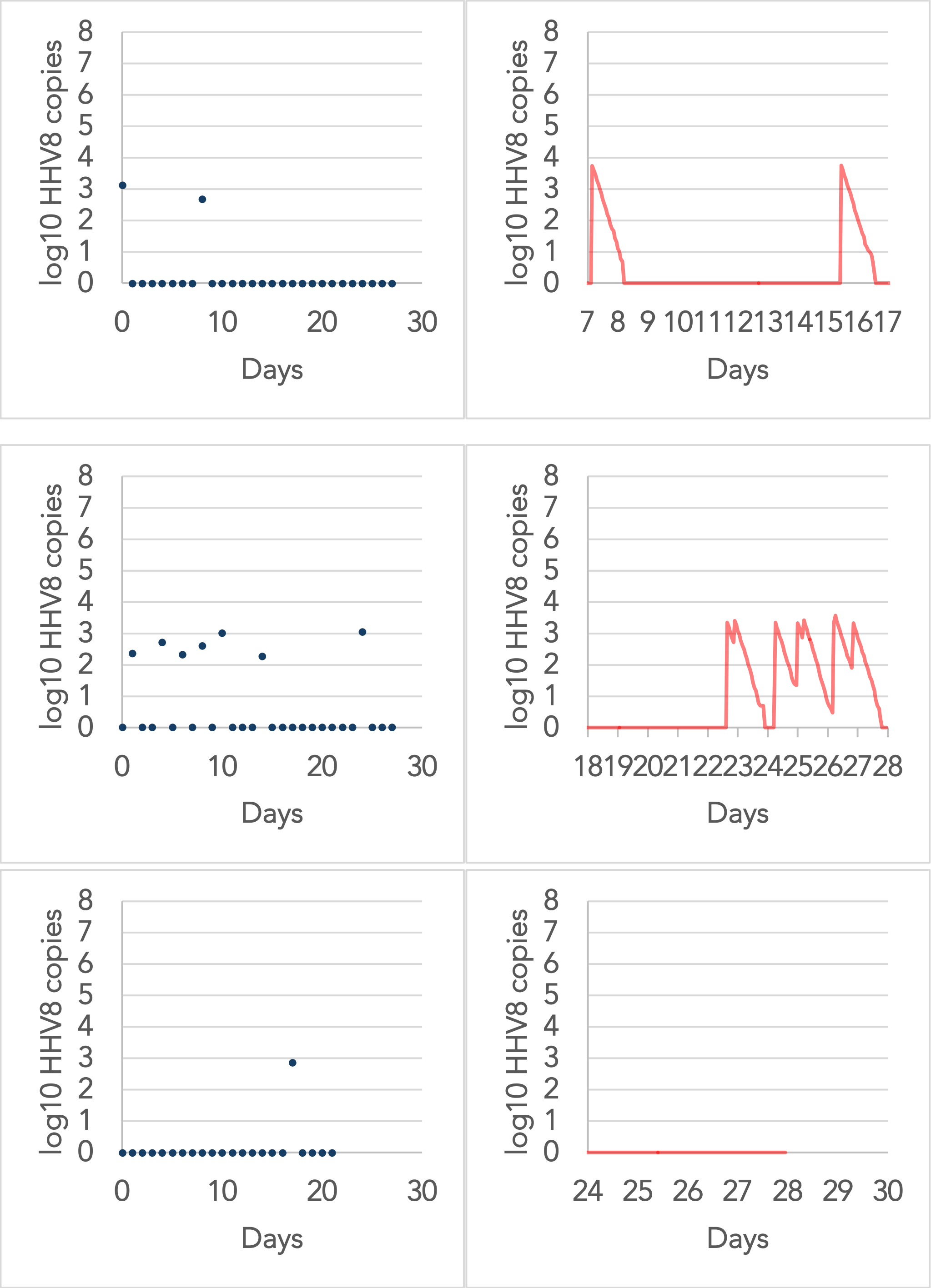

log10 HHV8 copies
8 7 6 5 4 3 2 1 0
0 10 20 30
Days
log10 HHV8 copies
8 7 6 5 4 3 2 1 0
7 8 9 10 11 12 13 14 15 16 17
Days
log10 HHV8 copies
8 7 6 5 4 3 2 1 0
0 10 20 30
Days
log10 HHV8 copies
8 7 6 5 4 3 2 1 0
18 19 20 21 22 23 24 25 26 27 28
Days
log10 HHV8 copies
8 7 6 5 4 3 2 1 0
0 10 20 30
Days
log10 HHV8 copies
8 7 6 5 4 3 2 1 0
24 25 26 27 28 29 30
Days

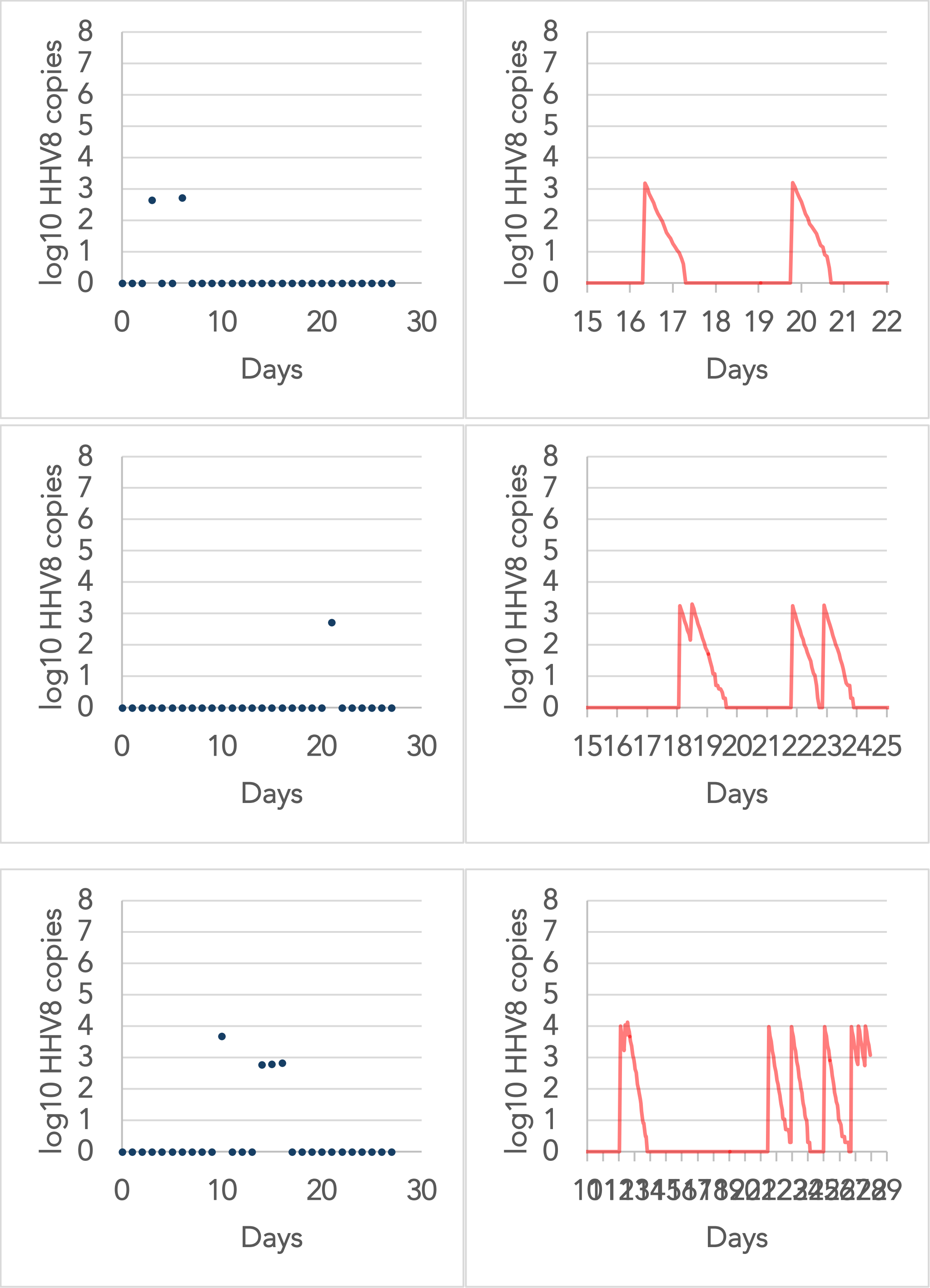
log10 HHV8 copies
8 7 6 5 4 3 2 1 0
0 10 20 30
Days
log10 HHV8 copies
8 7 6 5 4 3 2 1 0
15 16 17 18 19 20 21 22
Days
log10 HHV8 copies
8 7 6 5 4 3 2 1 0
0 10 20 30
Days
log10 HHV8 copies
8 7 6 5 4 3 2 1 0
Days
log10 HHV8 copies
8 7 6 5 4 3 2 1 0
0 10 20 30
Days
log10 HHV8 copies
8 7 6 5 4 3 2 1 0
Days

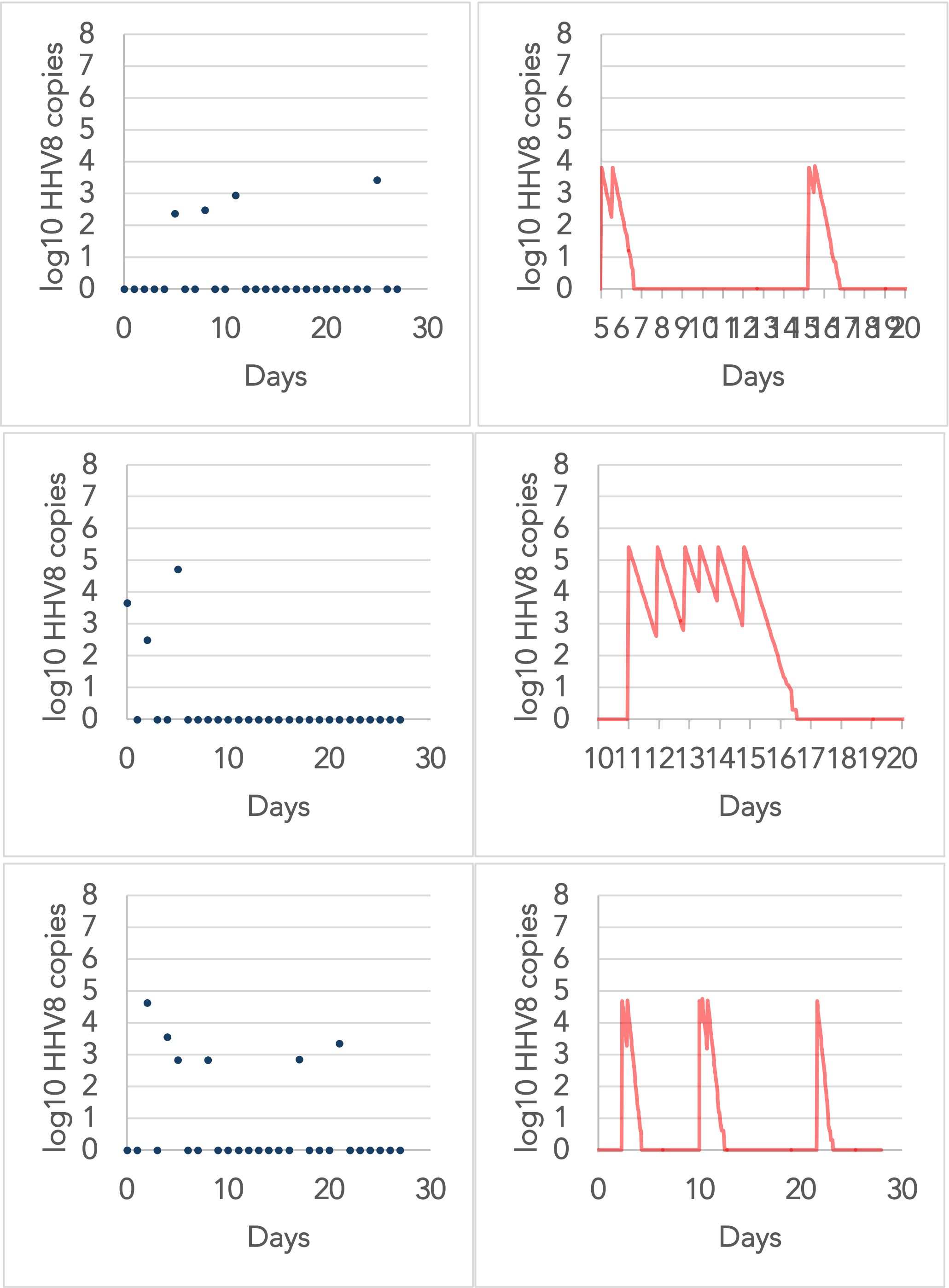
log10 HHV8 copies
Days
log10 HHV8 copies
Days
log10 HHV8 copies
Days
log10 HHV8 copies
Days
log10 HHV8 copies
Days
log10 HHV8 copies
Days

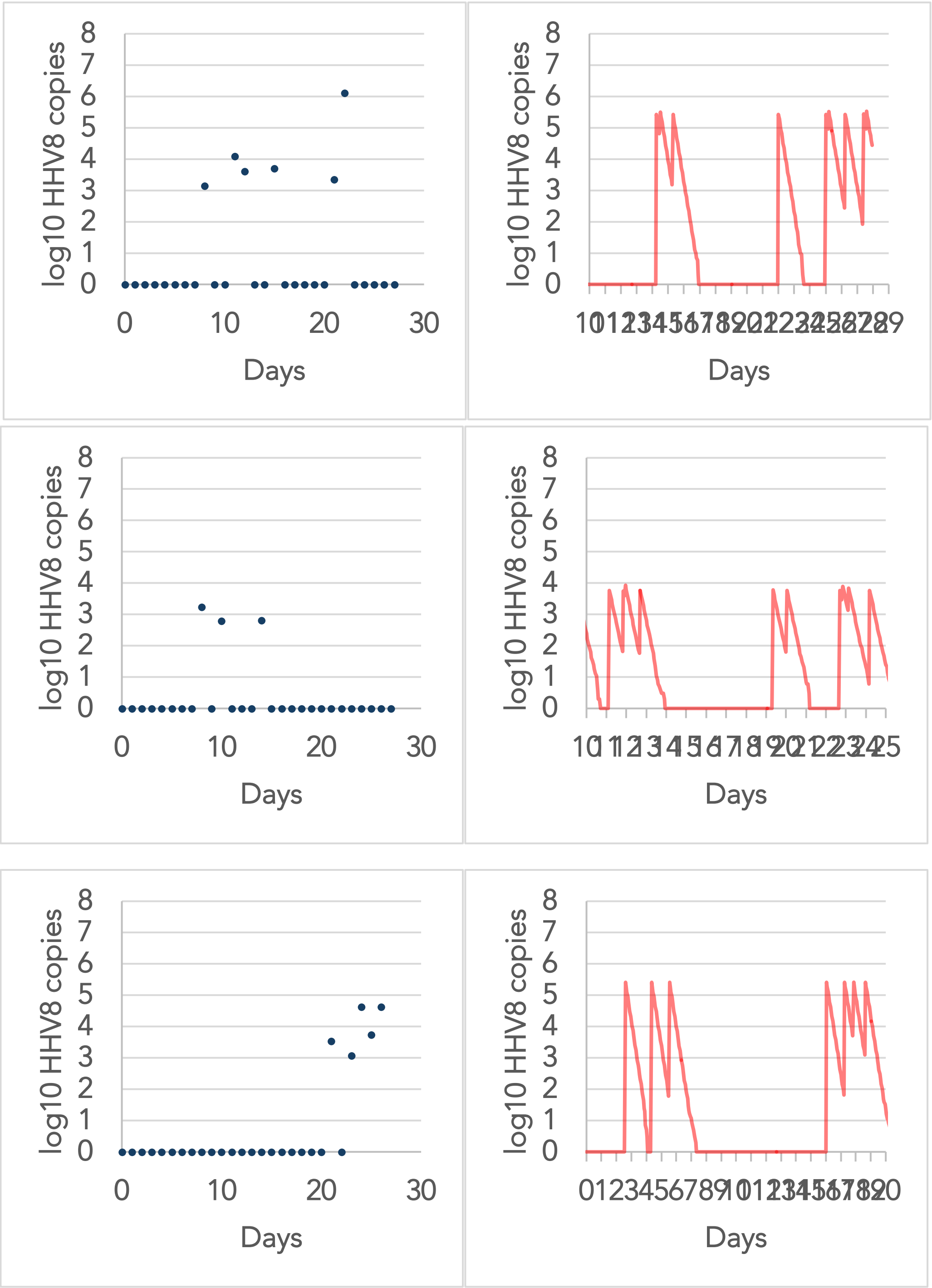

log10 HHV8 copies
0 1 2 3 4 5 6 7 8
0 10 20 30
Days
log10 HHV8 copies
0 1 2 3 4 5 6 7 8
Days
log10 HHV8 copies
0 1 2 3 4 5 6 7 8
0 10 20 30
Days
log10 HHV8 copies
0 1 2 3 4 5 6 7 8
Days
log10 HHV8 copies
0 1 2 3 4 5 6 7 8
0 10 20 30
Days
log10 HHV8 copies
0 1 2 3 4 5 6 7 8
Days

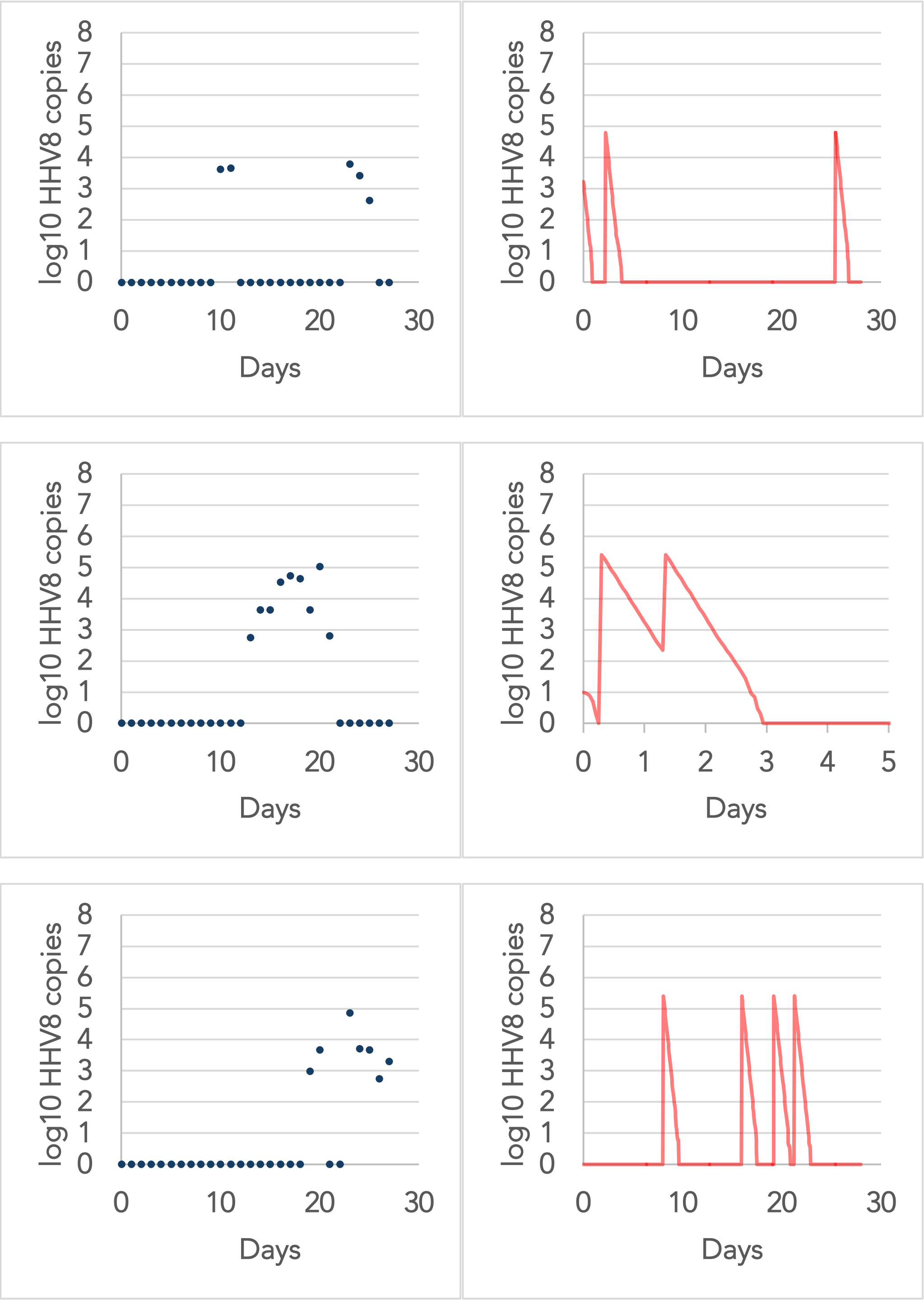
log10 HHV8 copies
Days
0
10
20
30
0
1
2
3
4
5
6
7
8
1
2
3
4
5

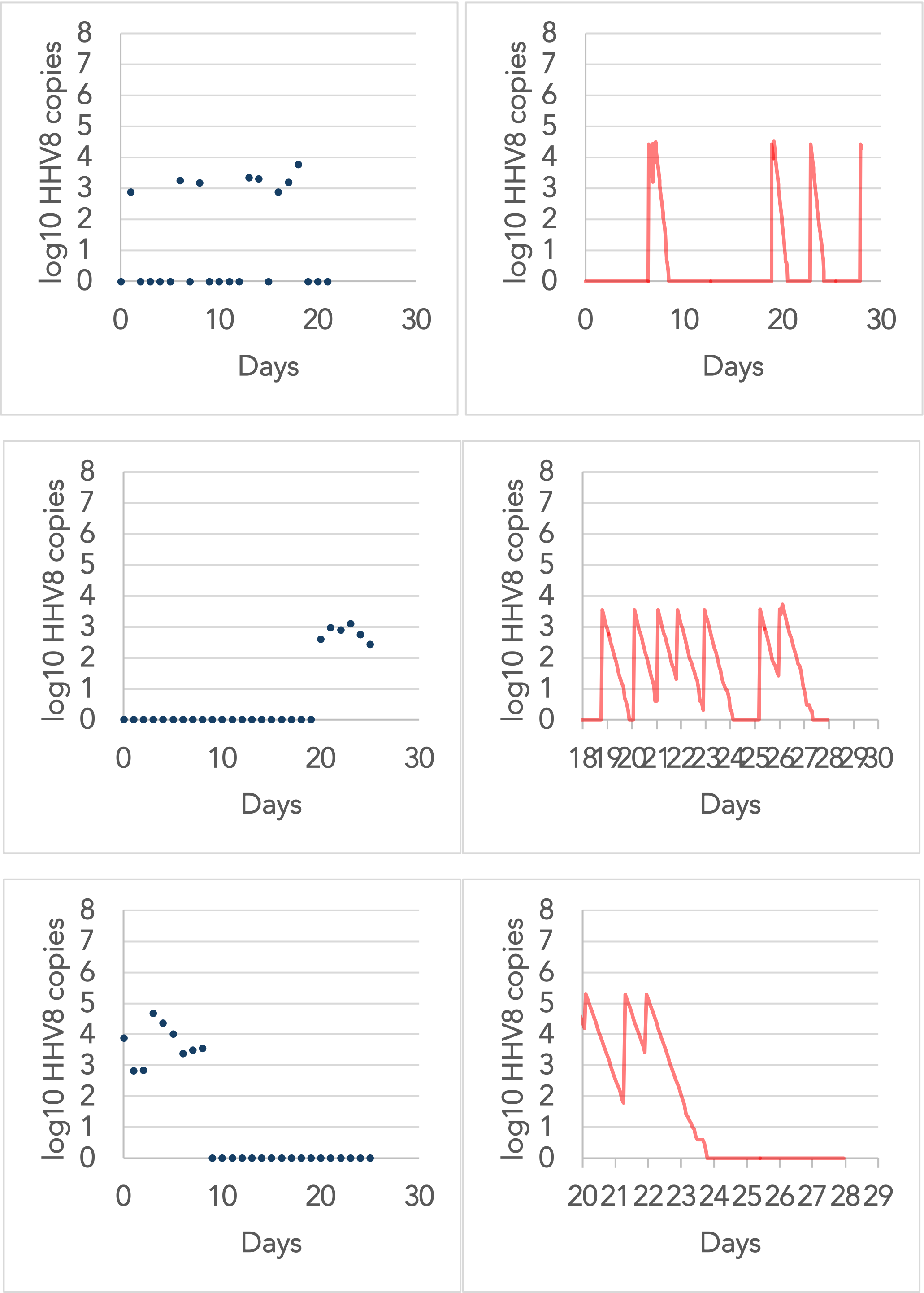
log10 HHV8 copies
Days

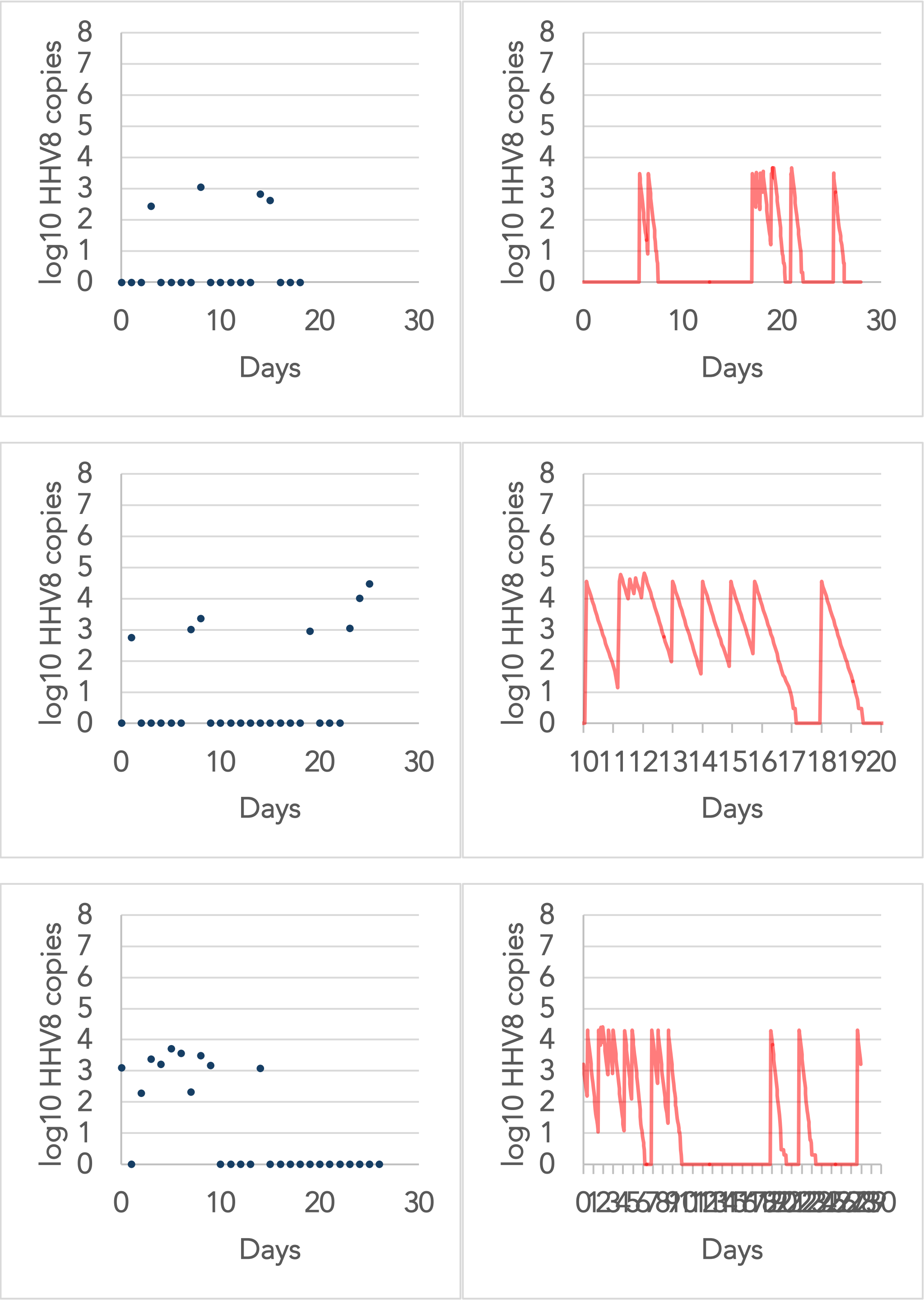
log10 HHV8 copies
Days
log10 HHV8 copies
Days
log10 HHV8 copies
Days
log10 HHV8 copies
Days
log10 HHV8 copies
Days
log10 HHV8 copies
Days

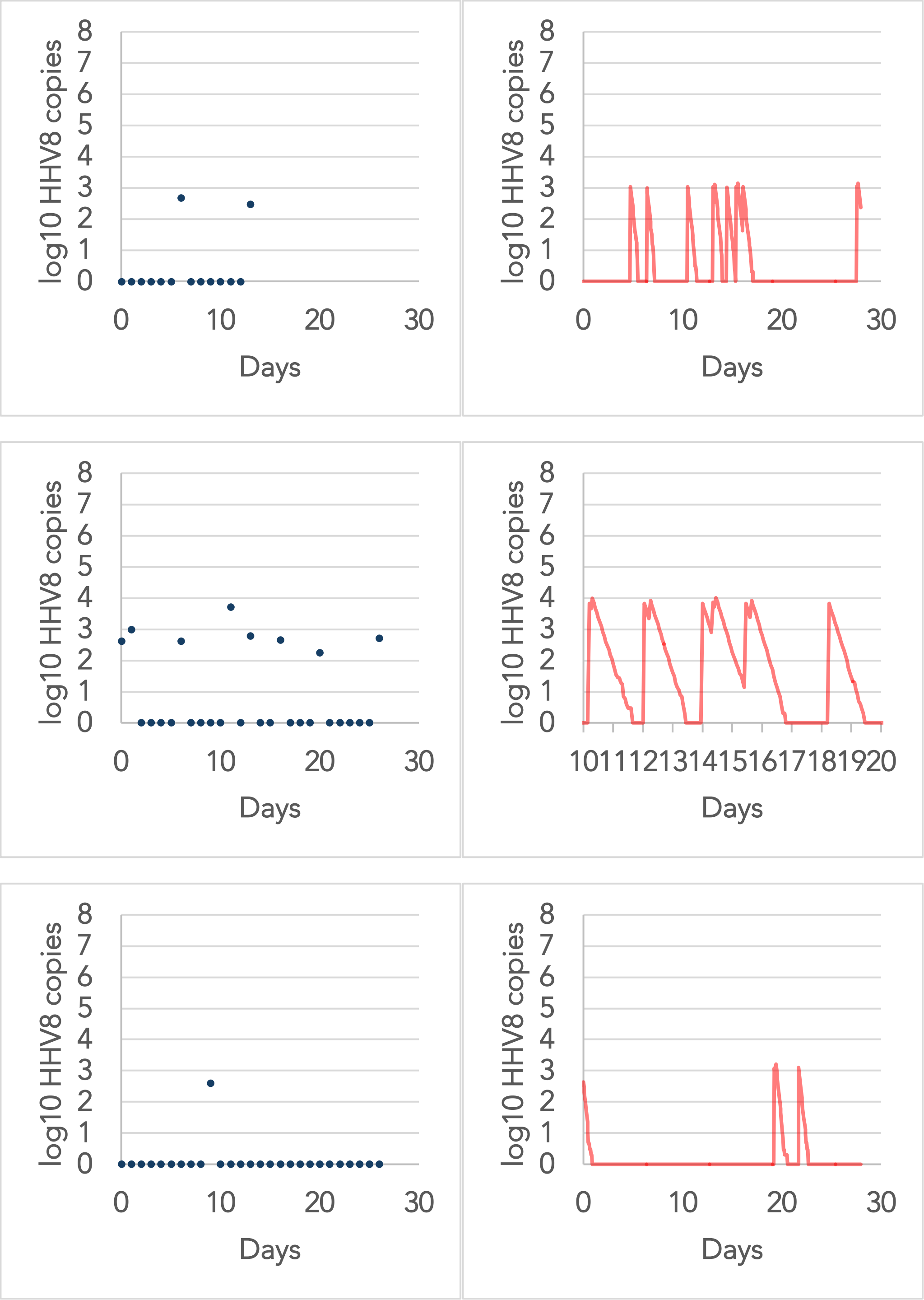
log10 HHV8 copies
0 1 2 3 4 5 6 7 8
0 10 20 30
Days
log10 HHV8 copies
0 1 2 3 4 5 6 7 8
0 10 20 30
Days
log10 HHV8 copies
0 1 2 3 4 5 6 7 8
0 10 20 30
Days
log10 HHV8 copies
0 1 2 3 4 5 6 7 8
10 11 12 13 14 15 16 17 18 19 20
Days
log10 HHV8 copies
0 1 2 3 4 5 6 7 8
0 10 20 30
Days
log10 HHV8 copies
0 1 2 3 4 5 6 7 8
0 10 20 30
Days

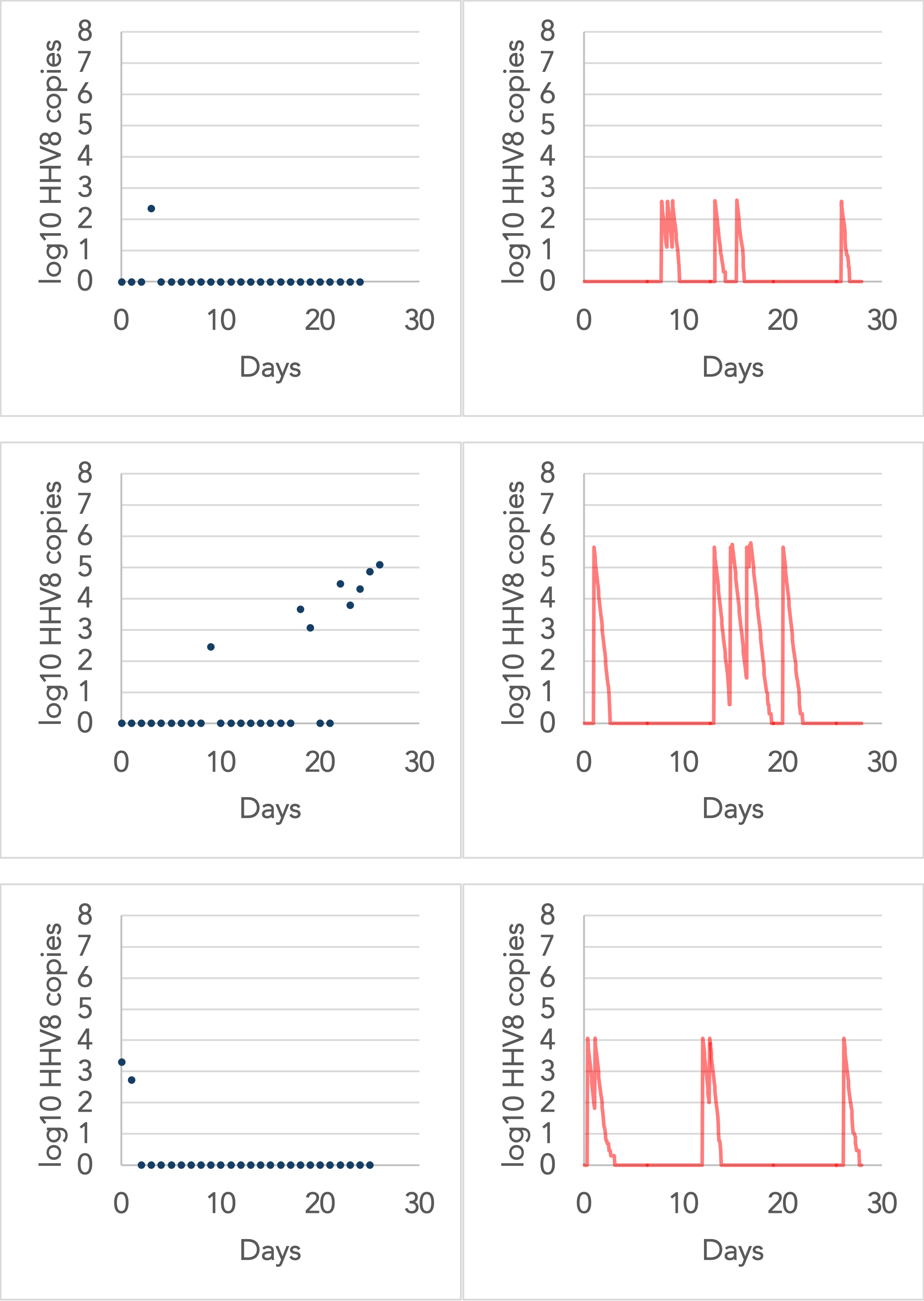
log10 HHV8 copies
0 1 2 3 4 5 6 7 8
0 10 20 30
Days

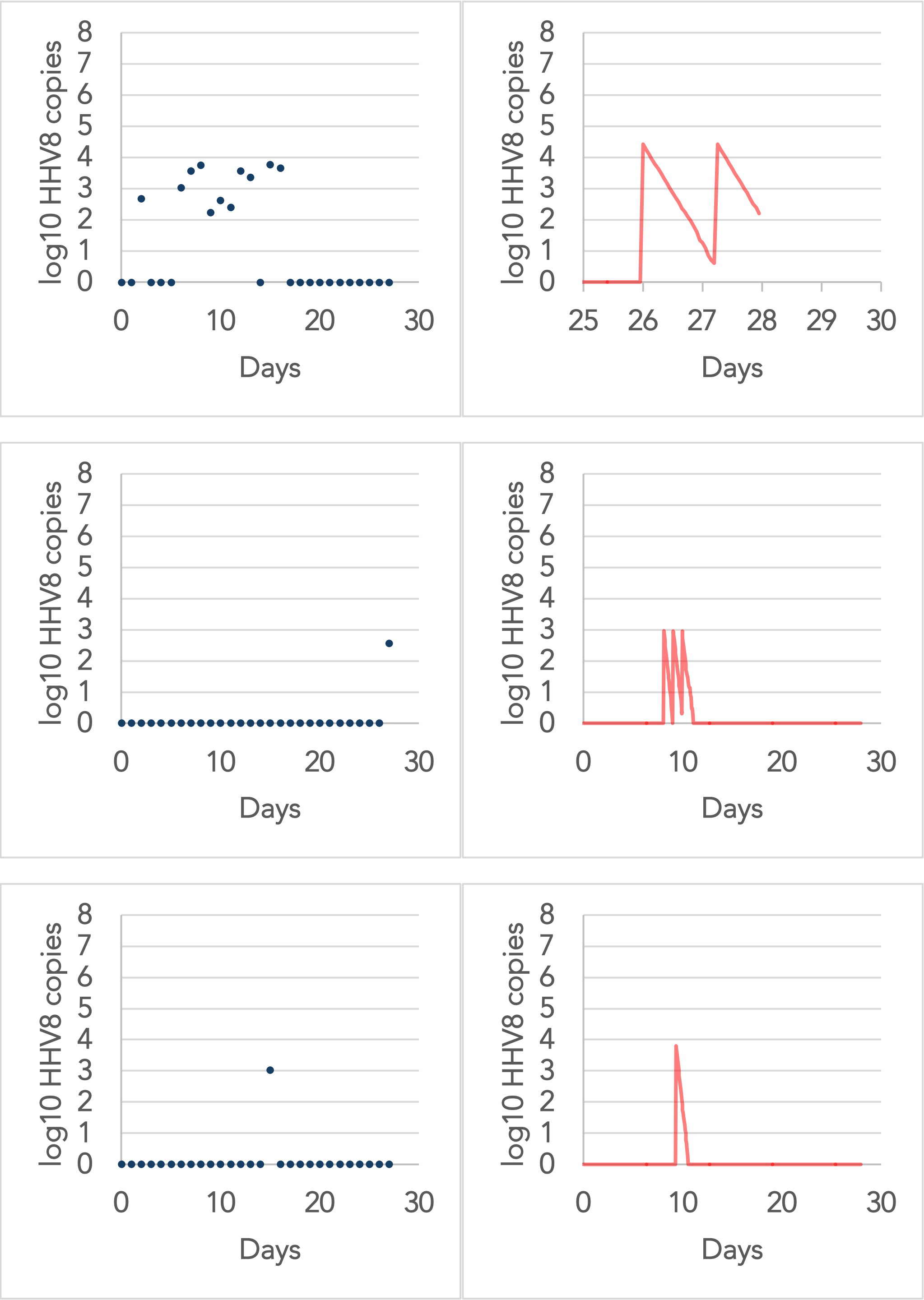
log10 HHV8 copies
Days
0
10
20
30
25
26
27
28
29
30
0
1
2
3
4
5
6
7
8

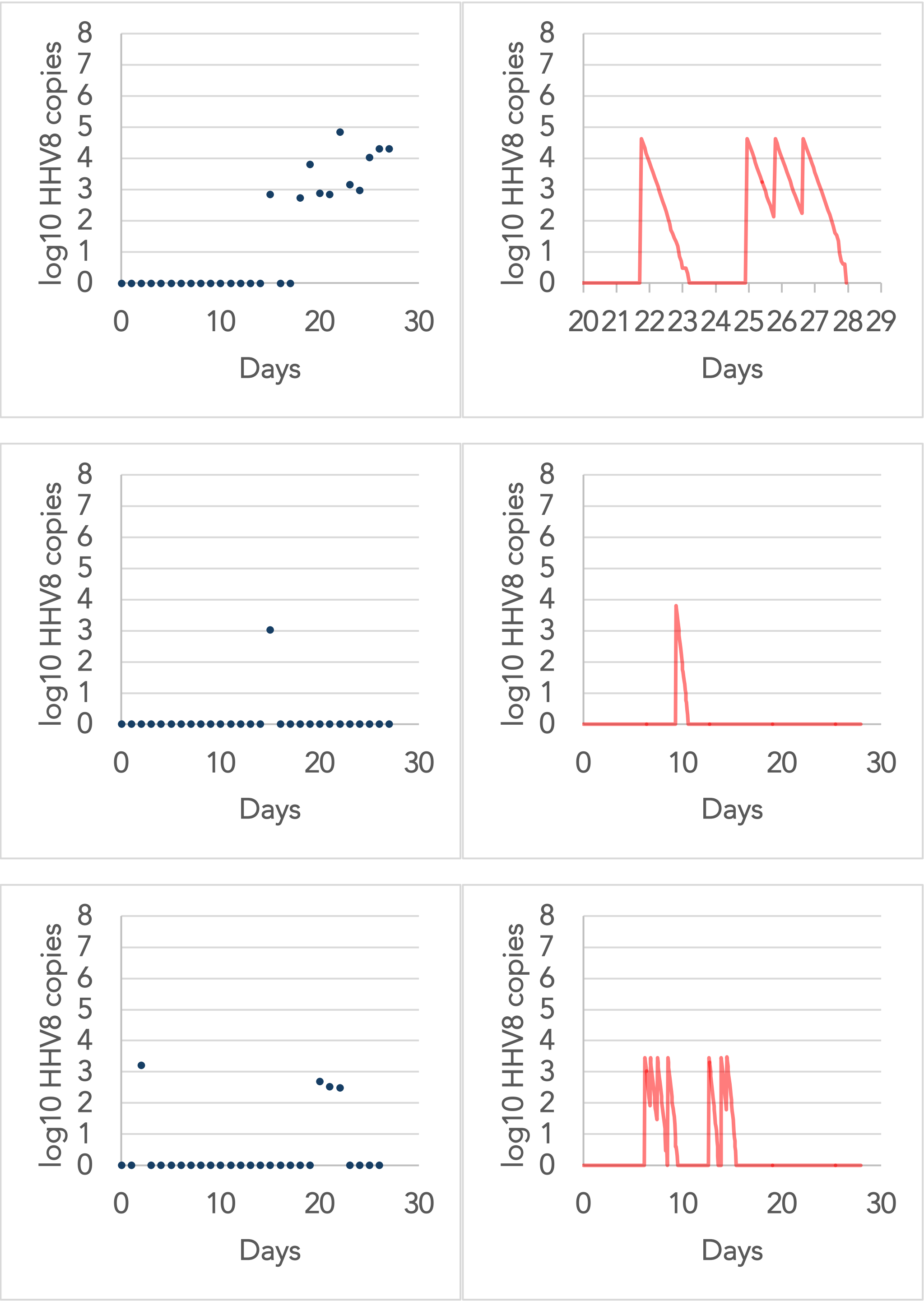

log10 HHV8 copies
0 1 2 3 4 5 6 7 8
0 10 20 30
Days
log10 HHV8 copies
0 1 2 3 4 5 6 7 8
20 21 22 23 24 25 26 27 28 29
Days
log10 HHV8 copies
0 1 2 3 4 5 6 7 8
0 10 20 30
Days
log10 HHV8 copies
0 1 2 3 4 5 6 7 8
0 10 20 30
Days
log10 HHV8 copies
0 1 2 3 4 5 6 7 8
0 10 20 30
Days
log10 HHV8 copies
0 1 2 3 4 5 6 7 8
0 10 20 30
Days

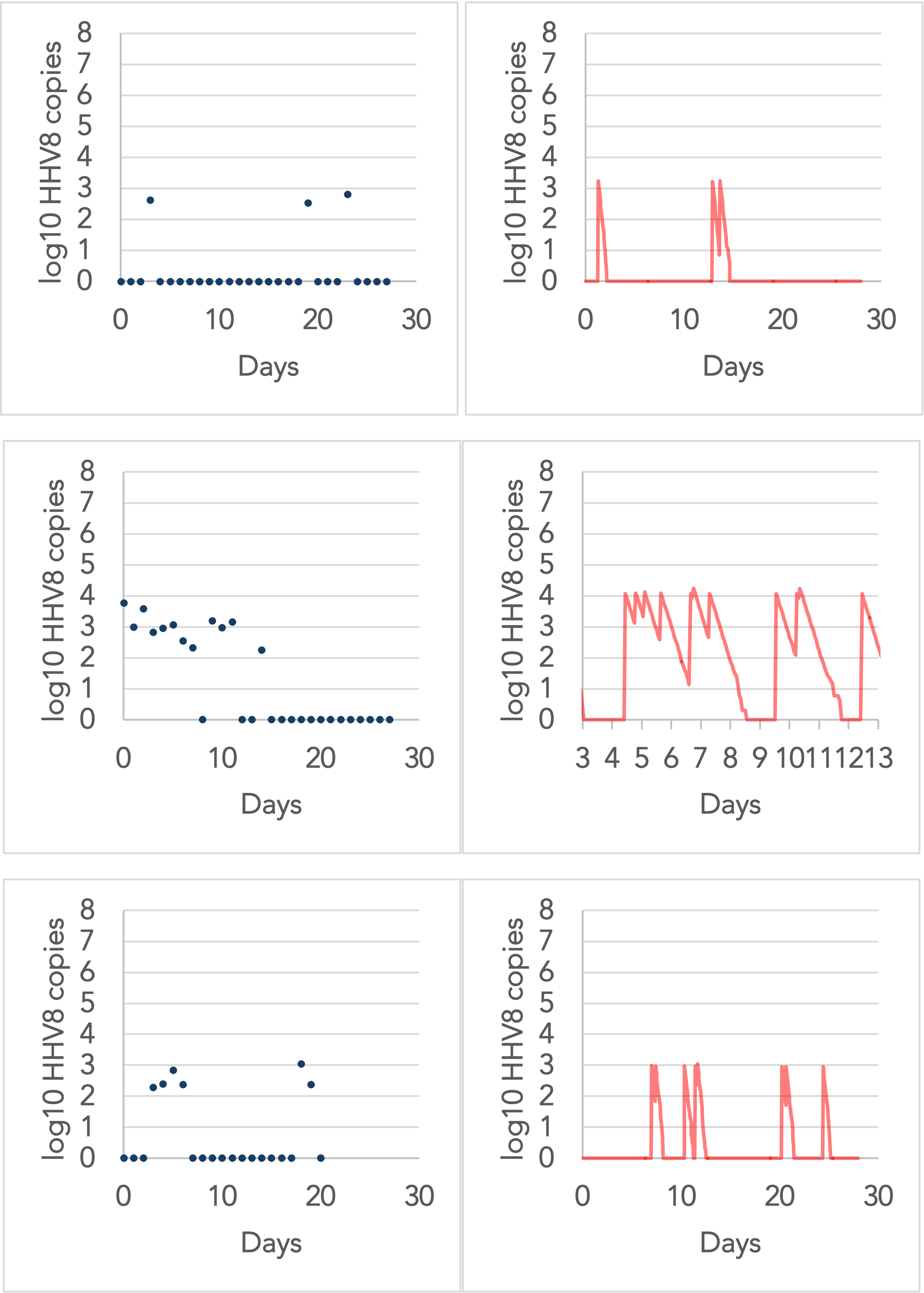
log10 HHV8 copies
Days

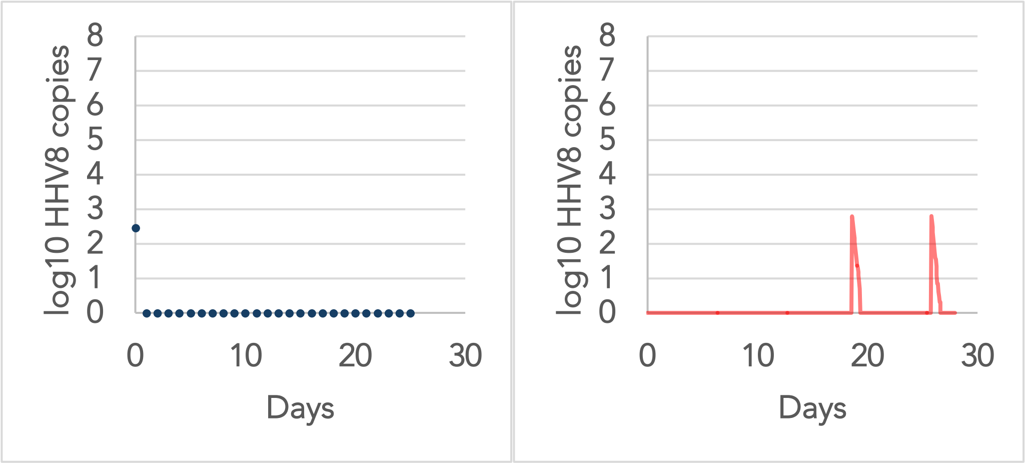

Figure S1. Diverse and accurate viral output from stochastic HHV8 model: examples of infrequent episodic shedding in 46 participants. Individual viral loads sampled daily from 10-30 days (blue dots) with stochastic model projections through 30 days (red lines, continuous sampling) using optimal parameter values for each individual. Model output is not intened to reproduce the timing and nature of each HHV8 reactivation but rather overall patterns of shedding. Summary statistics for model fitting were extracted from daily sampling of model data for fitting to data.

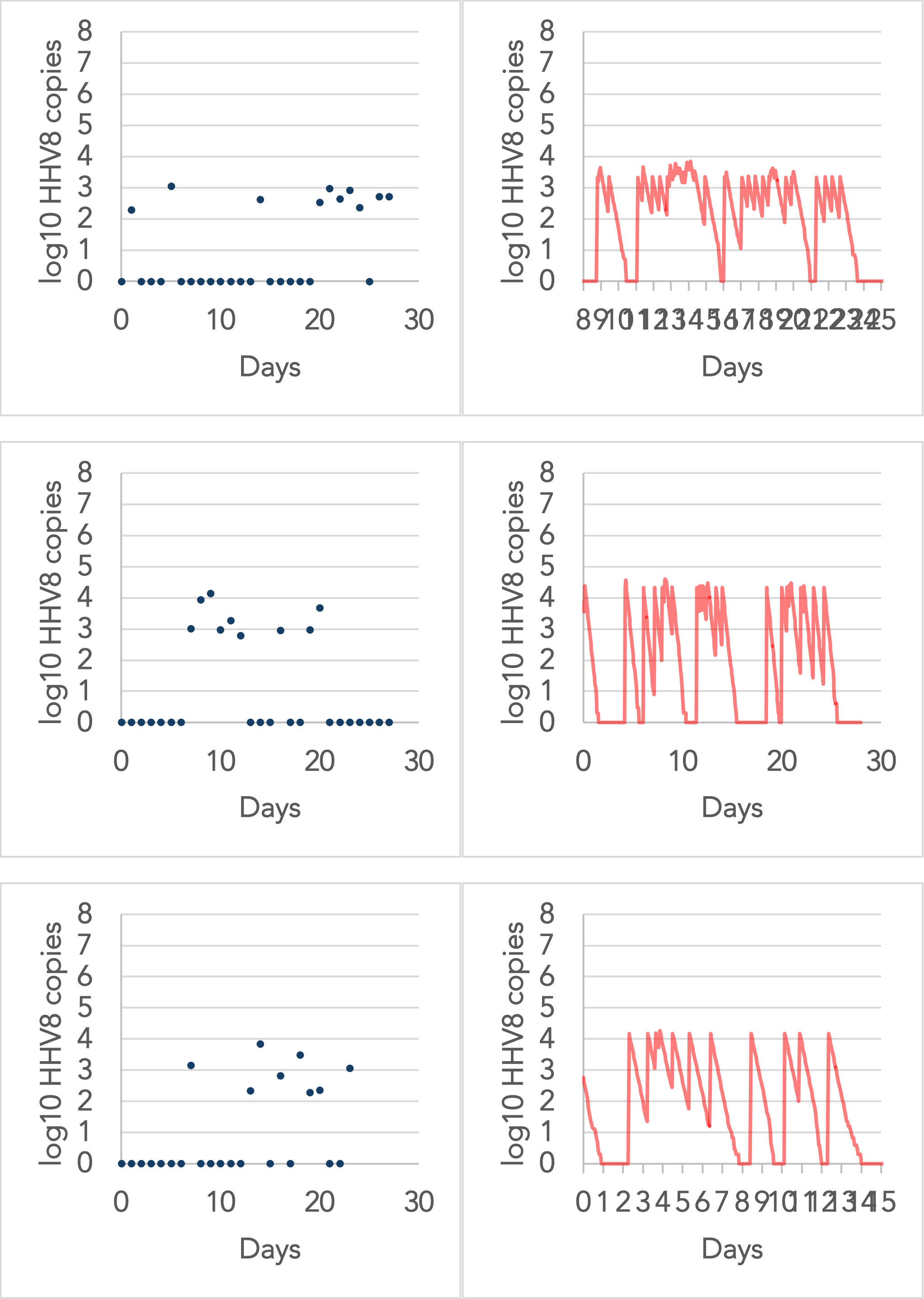
log10 HHV8 copies
Days
log10 HHV8 copies
Days
log10 HHV8 copies
Days
log10 HHV8 copies
Days
log10 HHV8 copies
Days
log10 HHV8 copies
Days

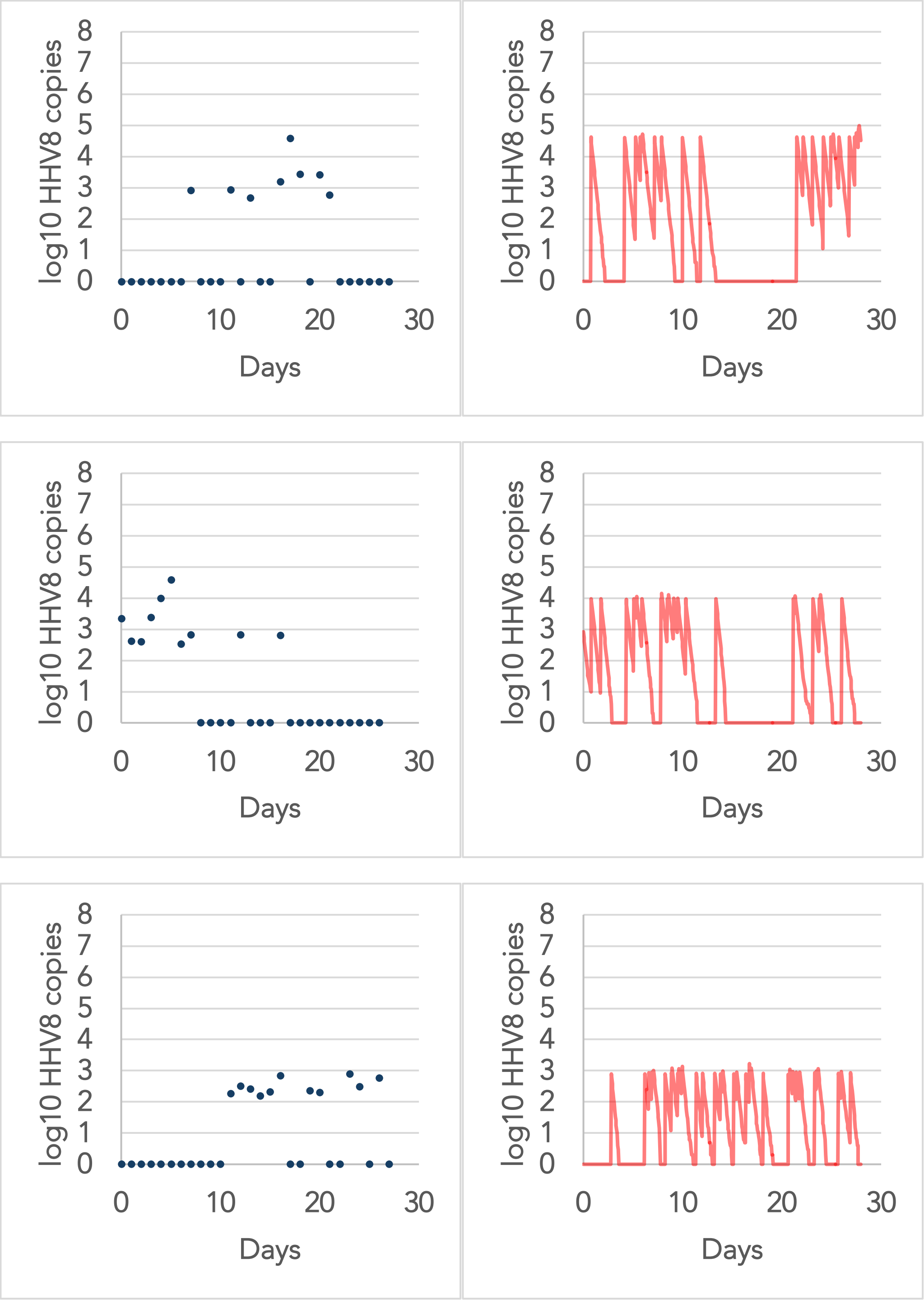
log10 HHV8 copies
0
1
2
3
4
5
6
7
8
0
10
20
30
Days

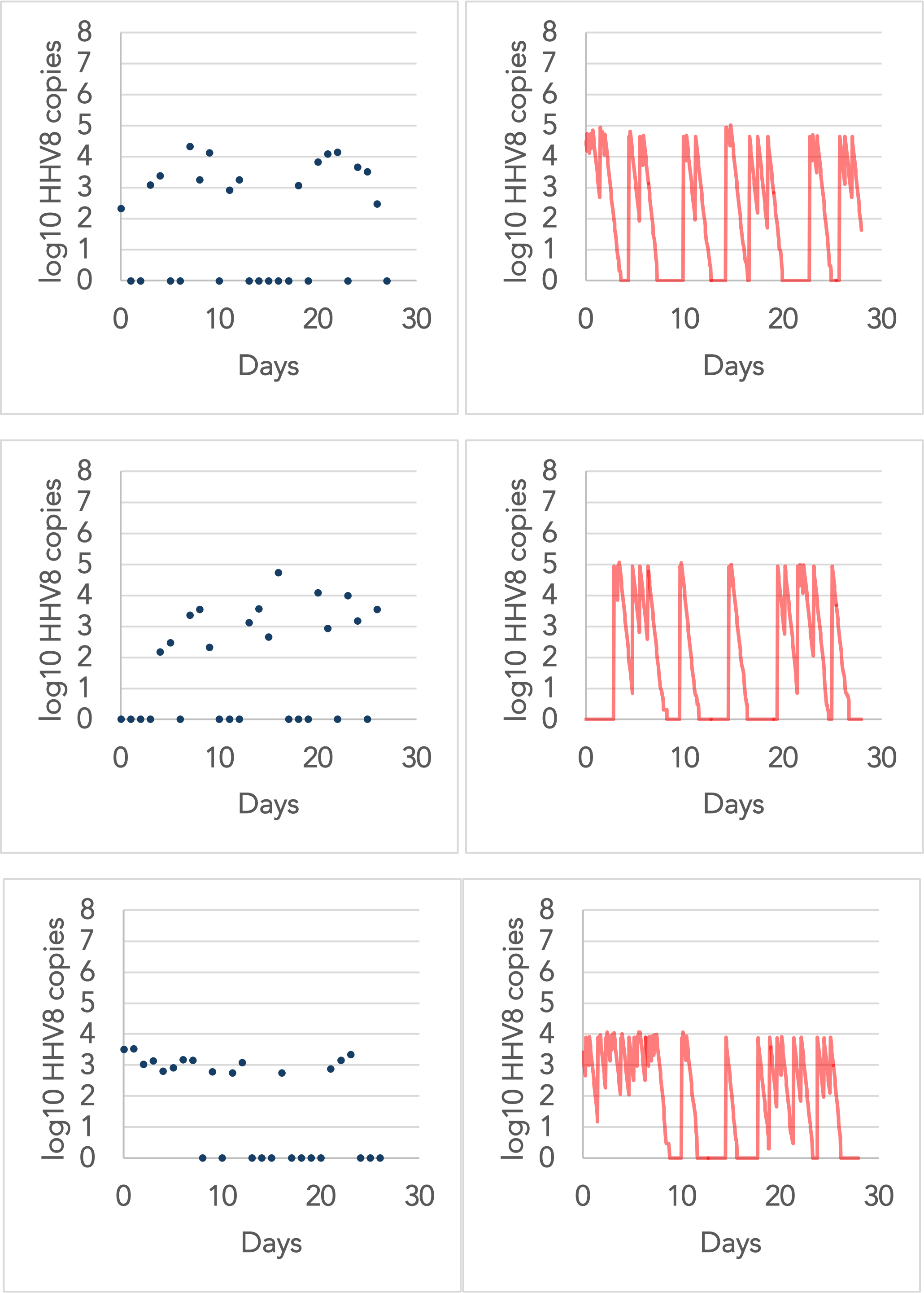
log10 HHV8 copies
Days
0
10
20
30
0
1
2
3
4
5
6
7
8

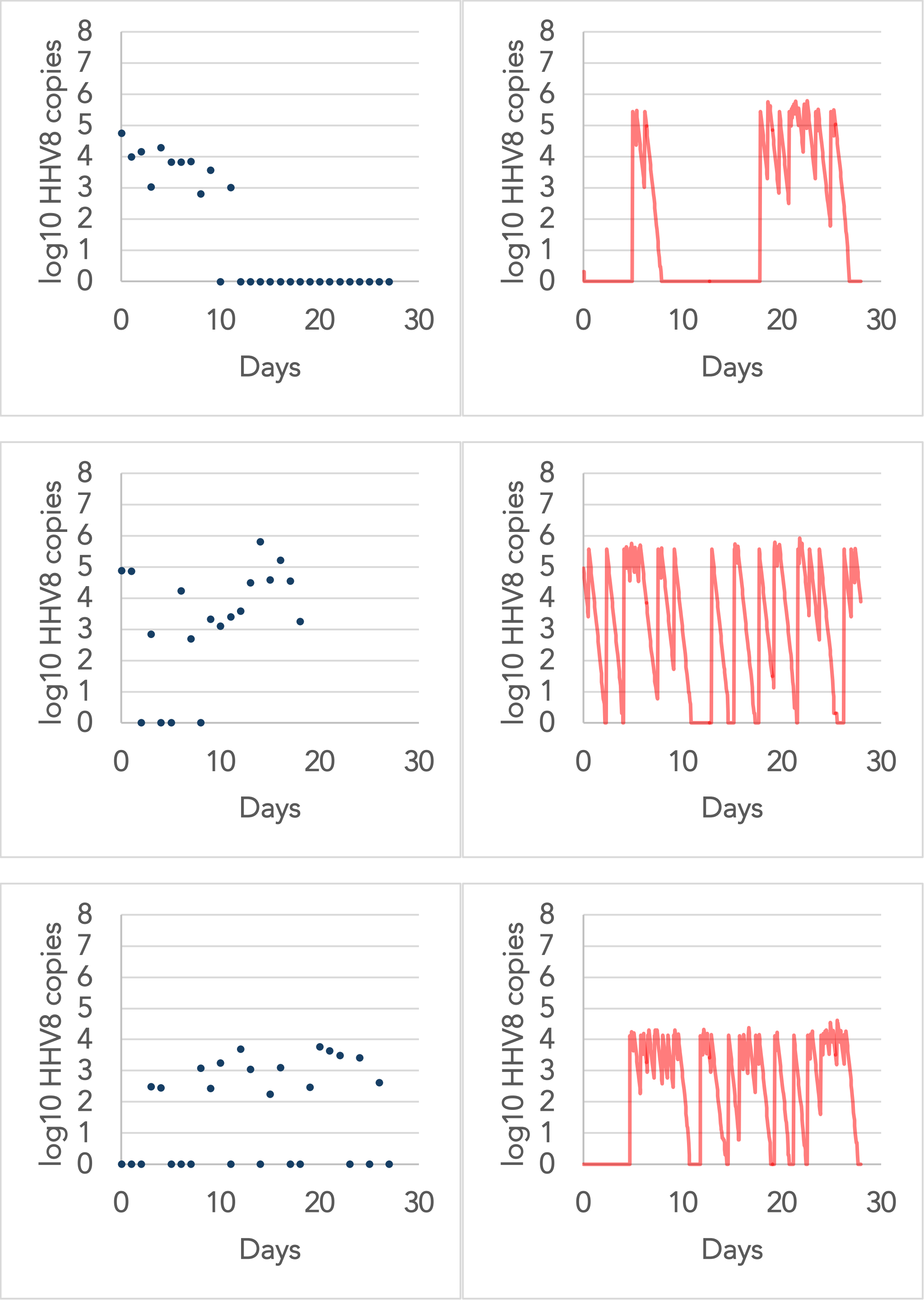

log10 HHV8 copies
8
7
6
5
4
3
2
1
0
0
10
20
30
Days
log10 HHV8 copies
8
7
6
5
4
3
2
1
0
0
10
20
30
Days
log10 HHV8 copies
8
7
6
5
4
3
2
1
0
0
10
20
30
Days
log10 HHV8 copies
8
7
6
5
4
3
2
1
0
0
10
20
30
Days
log10 HHV8 copies
8
7
6
5
4
3
2
1
0
0
10
20
30
Days
log10 HHV8 copies
8
7
6
5
4
3
2
1
0
0
10
20
30
Days

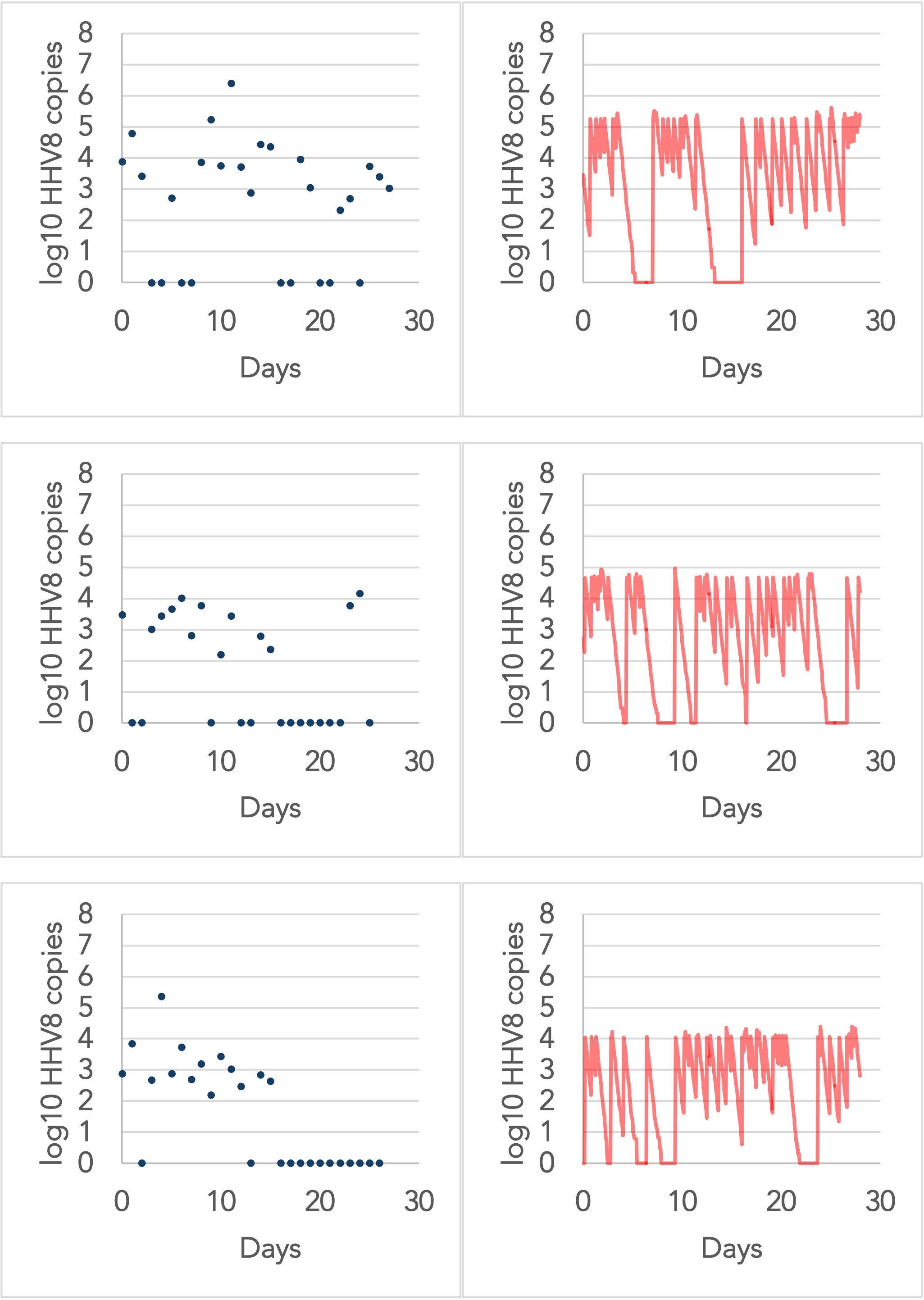
log10 HHV8 copies
Days
log10 HHV8 copies
Days
log10 HHV8 copies
Days
log10 HHV8 copies
Days
log10 HHV8 copies
Days
log10 HHV8 copies
Days

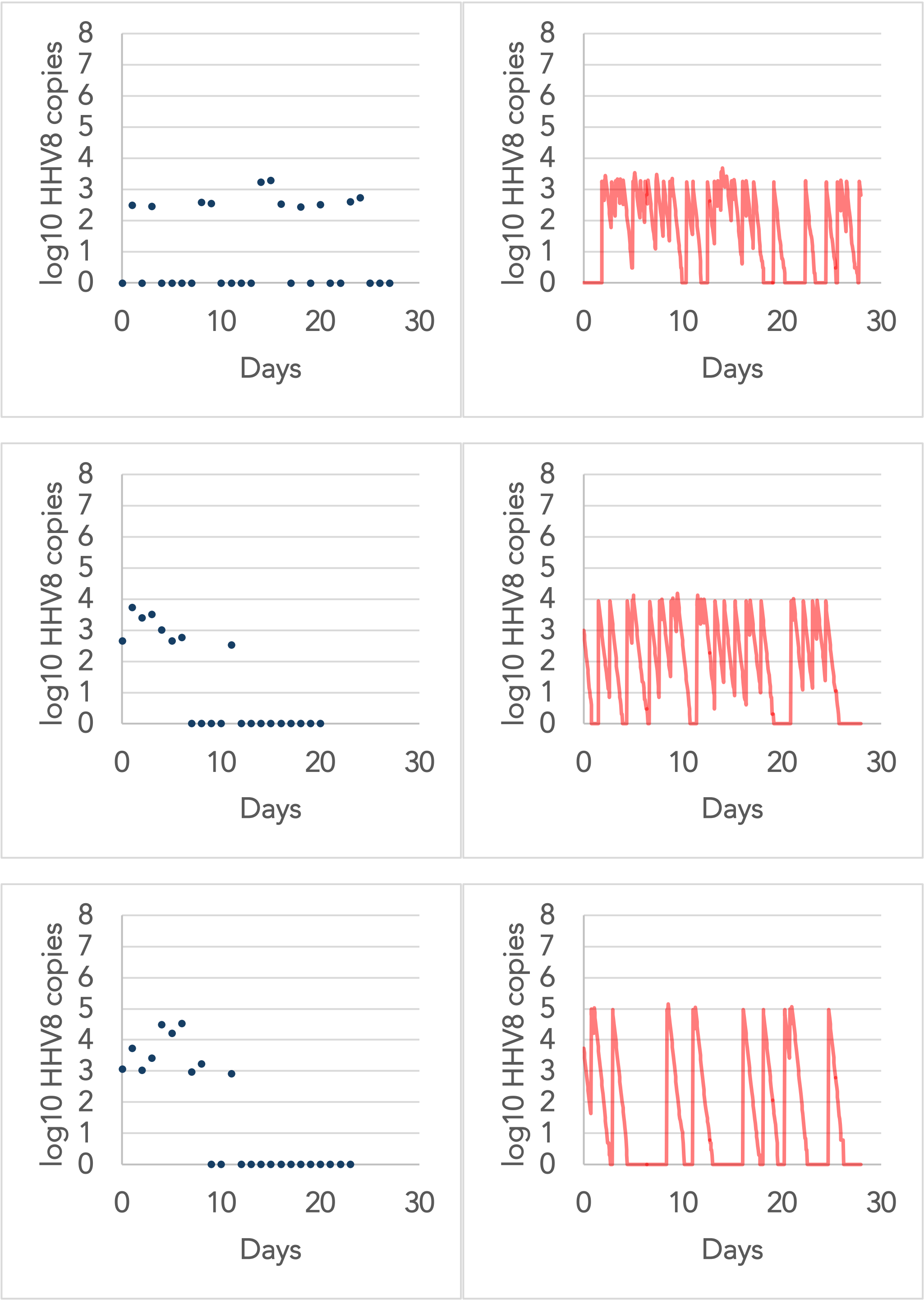
log10 HHV8 copies
8
7
6
5
4
3
2
1
0
0
10
20
30
Days
log10 HHV8 copies
8
7
6
5
4
3
2
1
0
0
10
20
30
Days
log10 HHV8 copies
8
7
6
5
4
3
2
1
0
0
10
20
30
Days
log10 HHV8 copies
8
7
6
5
4
3
2
1
0
0
10
20
30
Days
log10 HHV8 copies
8
7
6
5
4
3
2
1
0
0
10
20
30
Days
log10 HHV8 copies
8
7
6
5
4
3
2
1
0
0
10
20
30
Days

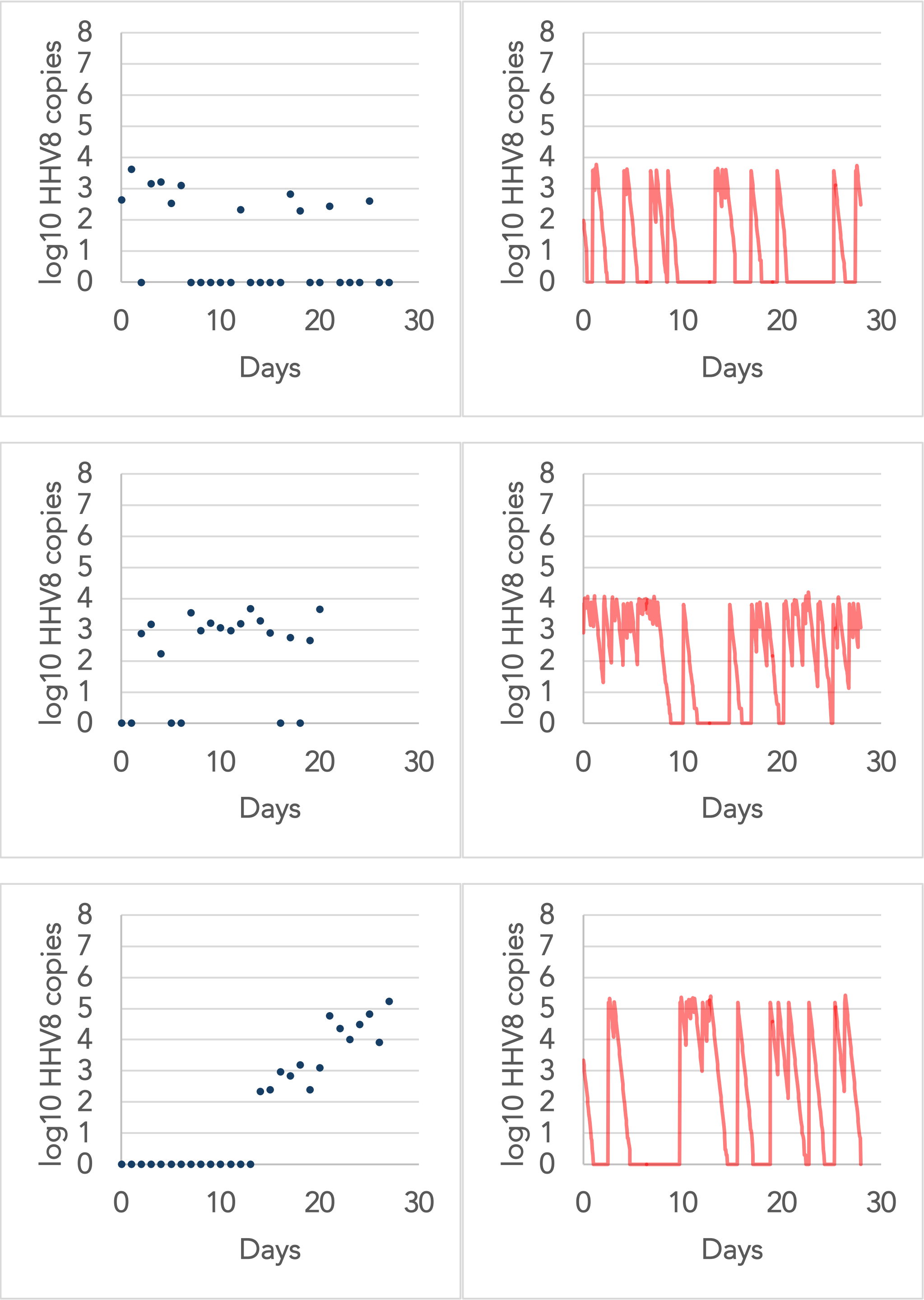

log10 HHV8 copies
0
1
2
3
4
5
6
7
8
0
10
20
30
Days

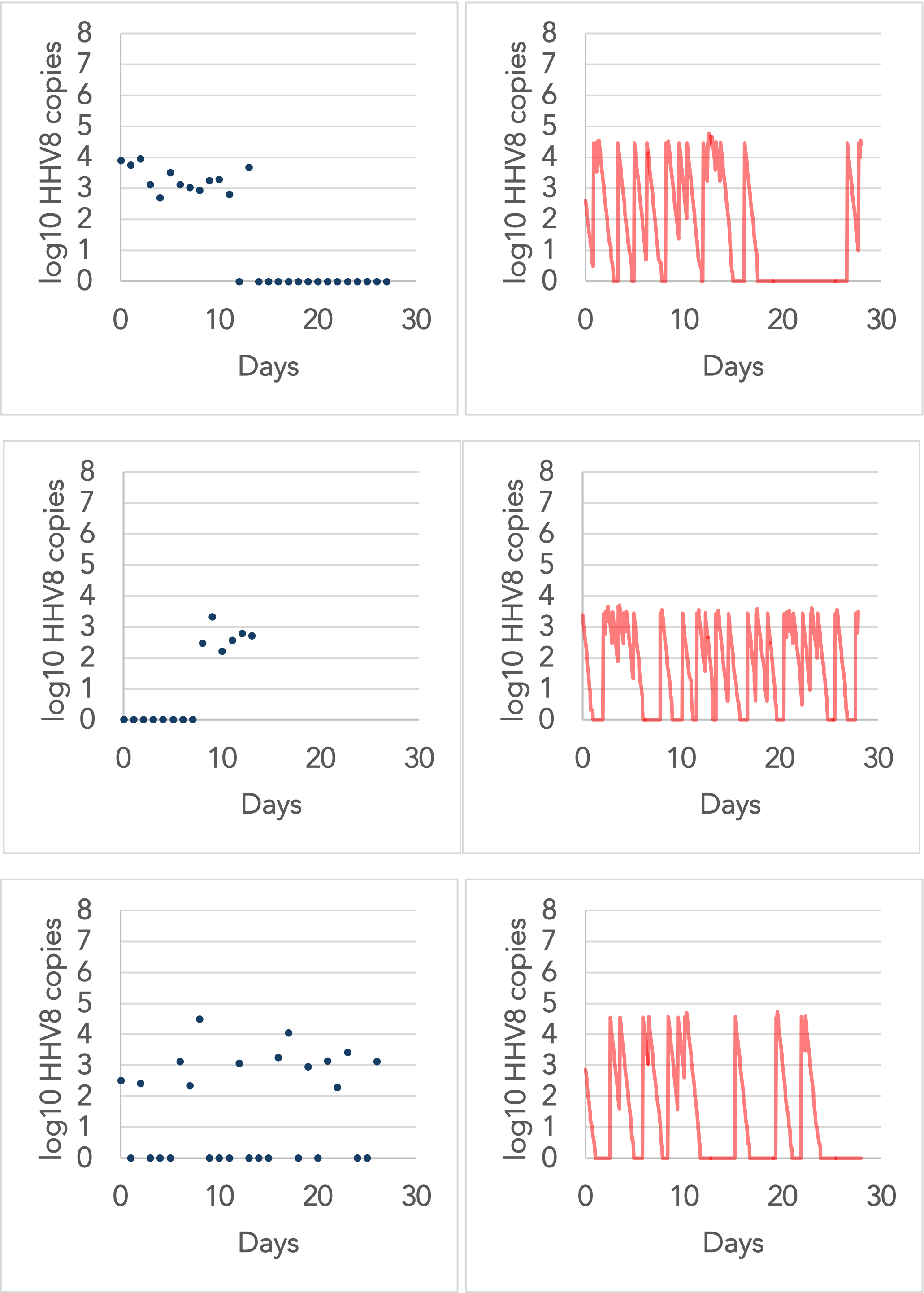
log10 HHV8 copies
8
7
6
5
4
3
2
1
0
0
10
20
30
Days
log10 HHV8 copies
8
7
6
5
4
3
2
1
0
0
10
20
30
Days
log10 HHV8 copies
8
7
6
5
4
3
2
1
0
0
10
20
30
Days
log10 HHV8 copies
8
7
6
5
4
3
2
1
0
0
10
20
30
Days
log10 HHV8 copies
8
7
6
5
4
3
2
1
0
0
10
20
30
Days
log10 HHV8 copies
8
7
6
5
4
3
2
1
0
0
10
20
30
Days

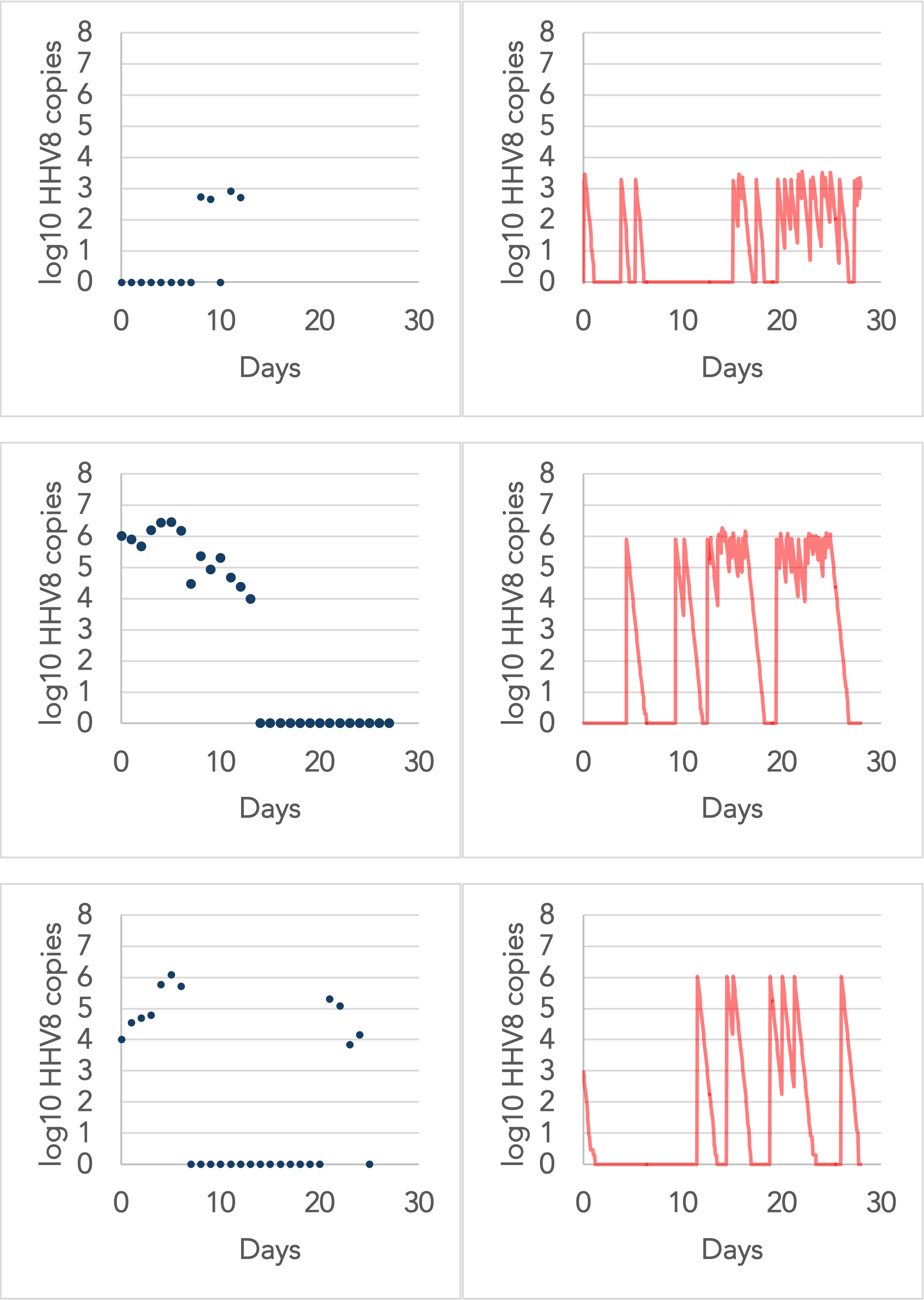
log10 HHV8 copies
Days
log10 HHV8 copies
Days
log10 HHV8 copies
Days
log10 HHV8 copies
Days
log10 HHV8 copies
Days
log10 HHV8 copies
Days

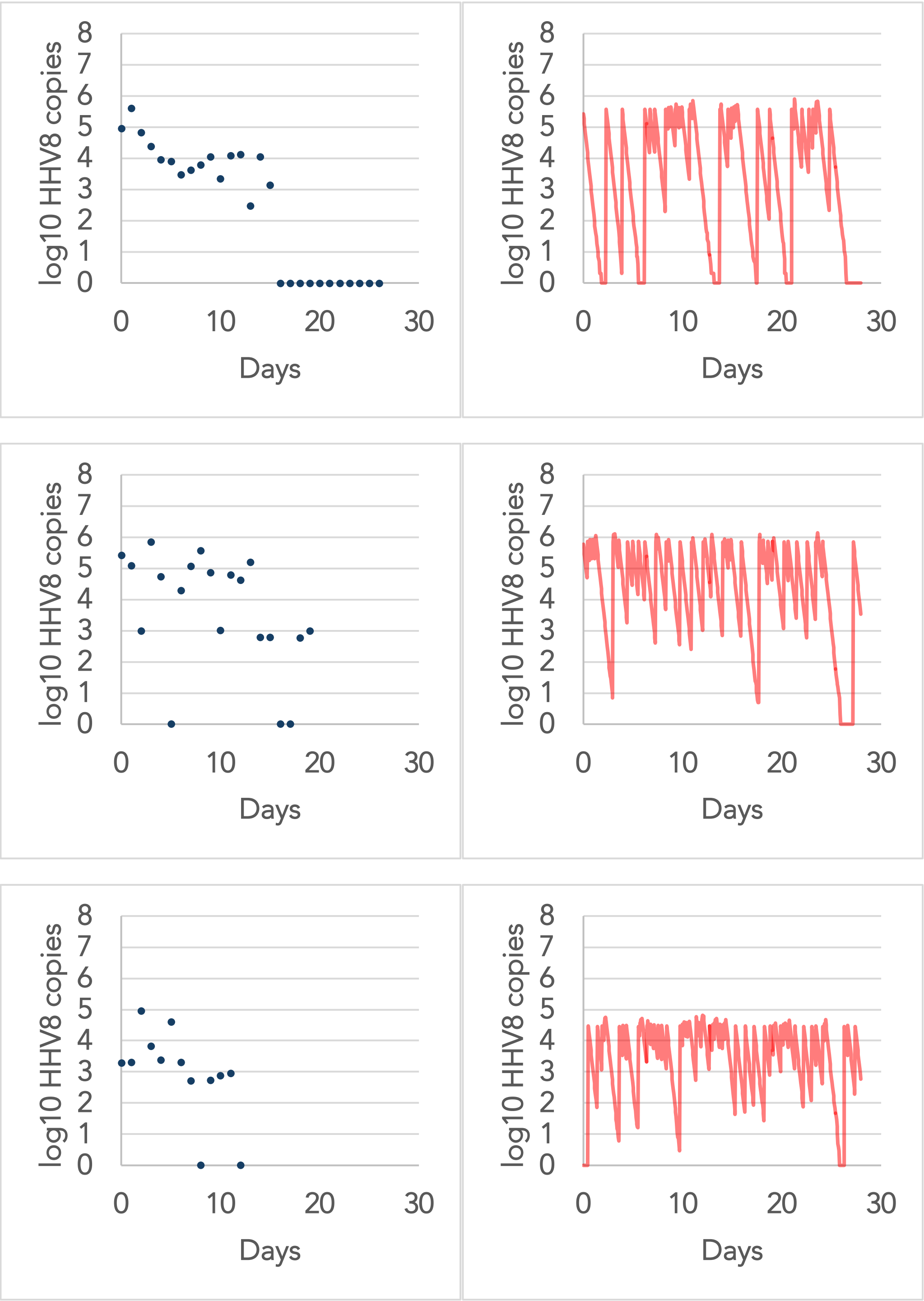
log10 HHV8 copies
0
1
2
3
4
5
6
7
8
0
10
20
30
Days

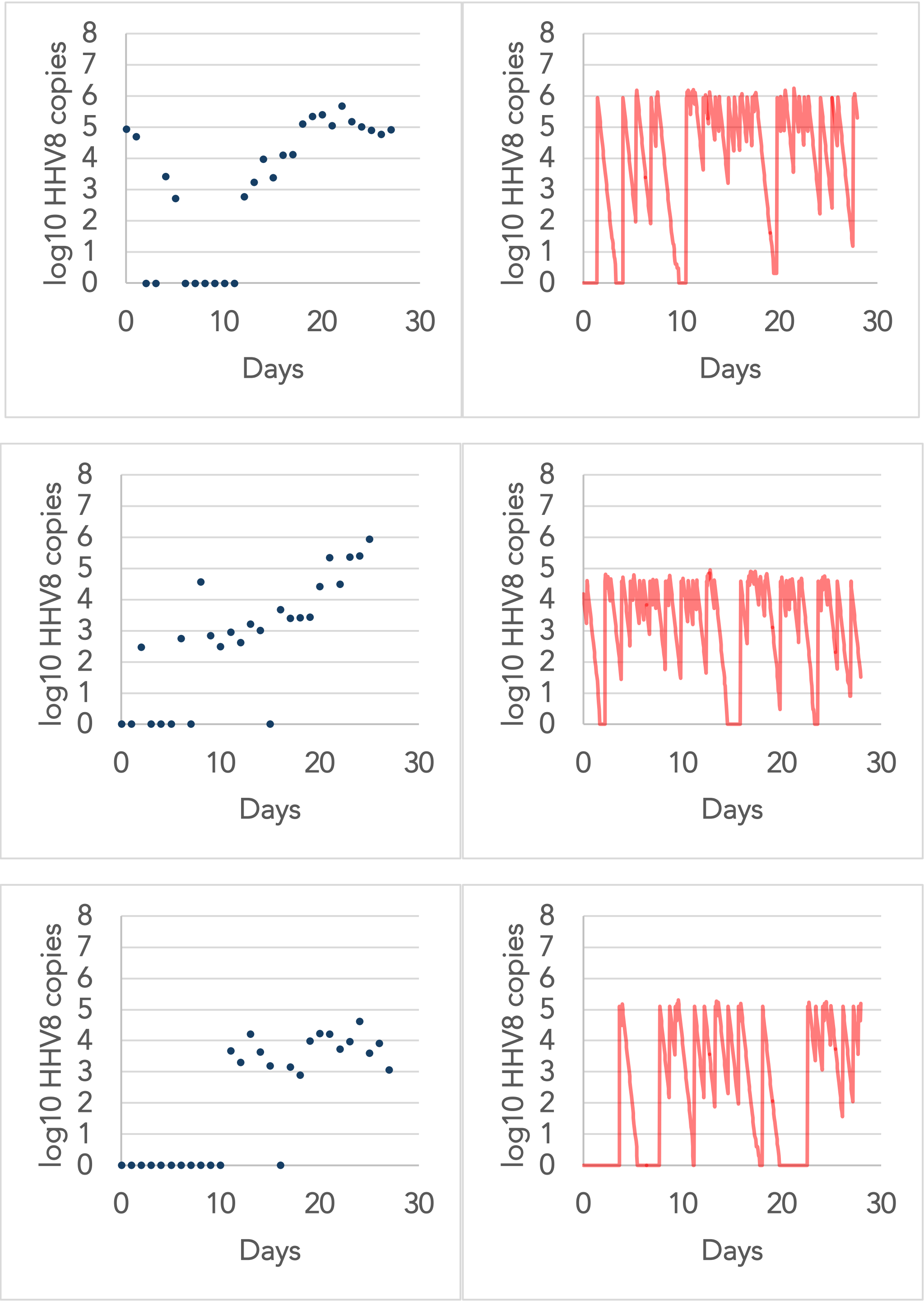
log10 HHV8 copies
Days
log10 HHV8 copies
Days
log10 HHV8 copies
Days
log10 HHV8 copies
Days
log10 HHV8 copies
Days
log10 HHV8 copies
Days

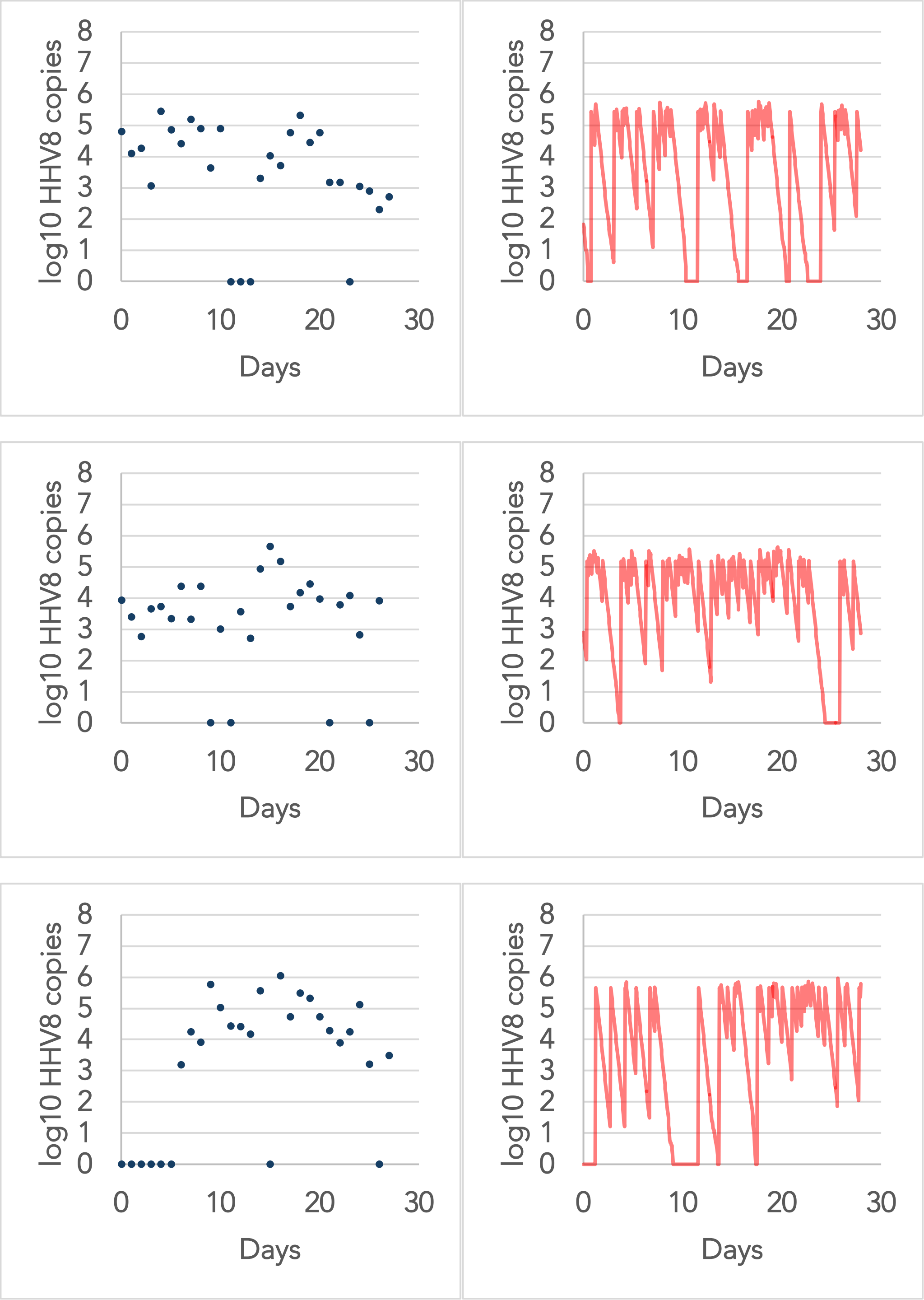
log10 HHV8 copies
0 1 2 3 4 5 6 7 8
0 10 20 30
Days

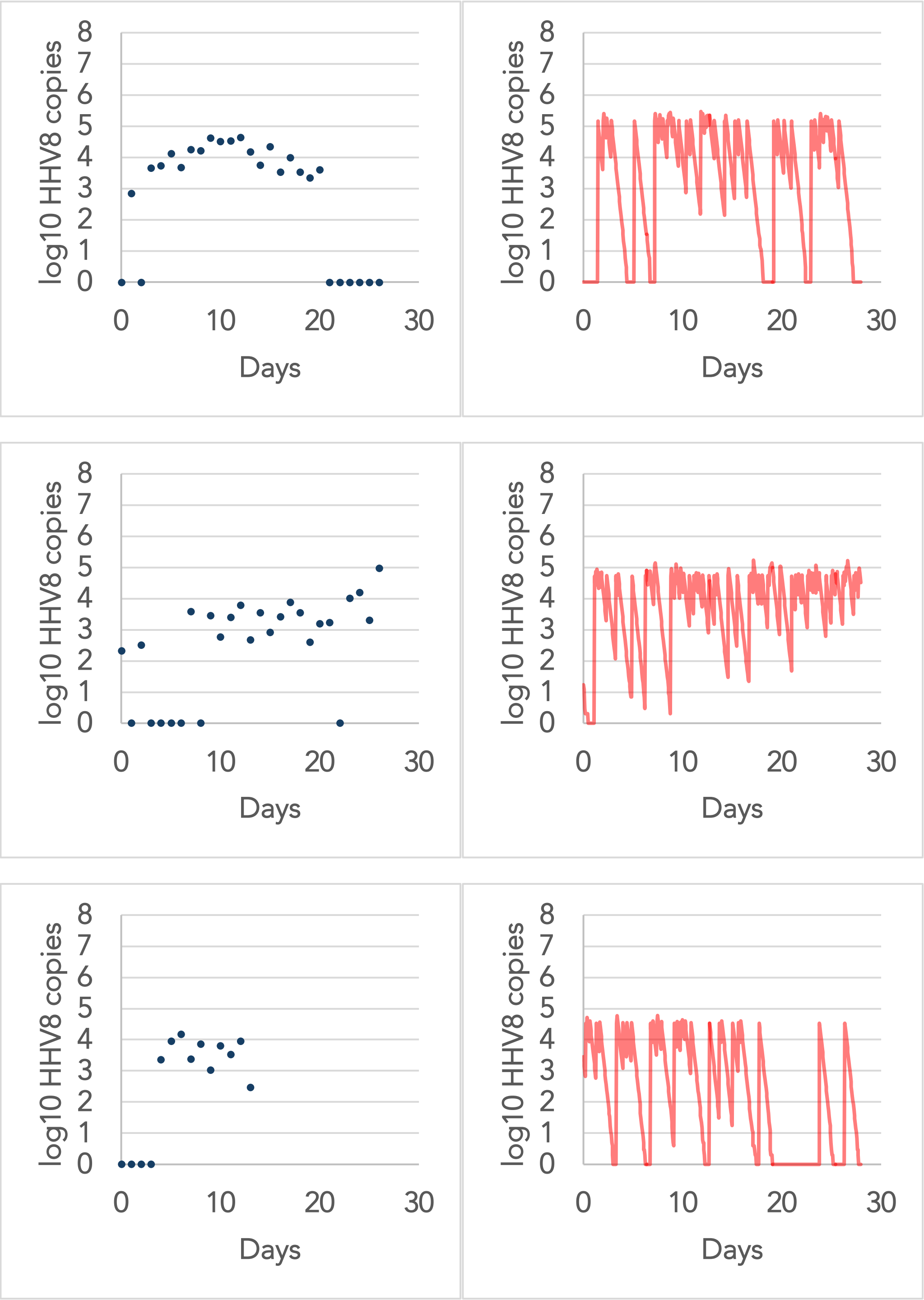
log10 HHV8 copies
0
1
2
3
4
5
6
7
8
0
10
20
30
Days
log10 HHV8 copies
0
1
2
3
4
5
6
7
8
0
10
20
30
Days
log10 HHV8 copies
0
1
2
3
4
5
6
7
8
0
10
20
30
Days
log10 HHV8 copies
0
1
2
3
4
5
6
7
8
0
10
20
30
Days
log10 HHV8 copies
0
1
2
3
4
5
6
7
8
0
10
20
30
Days
log10 HHV8 copies
0
1
2
3
4
5
6
7
8
0
10
20
30
Days

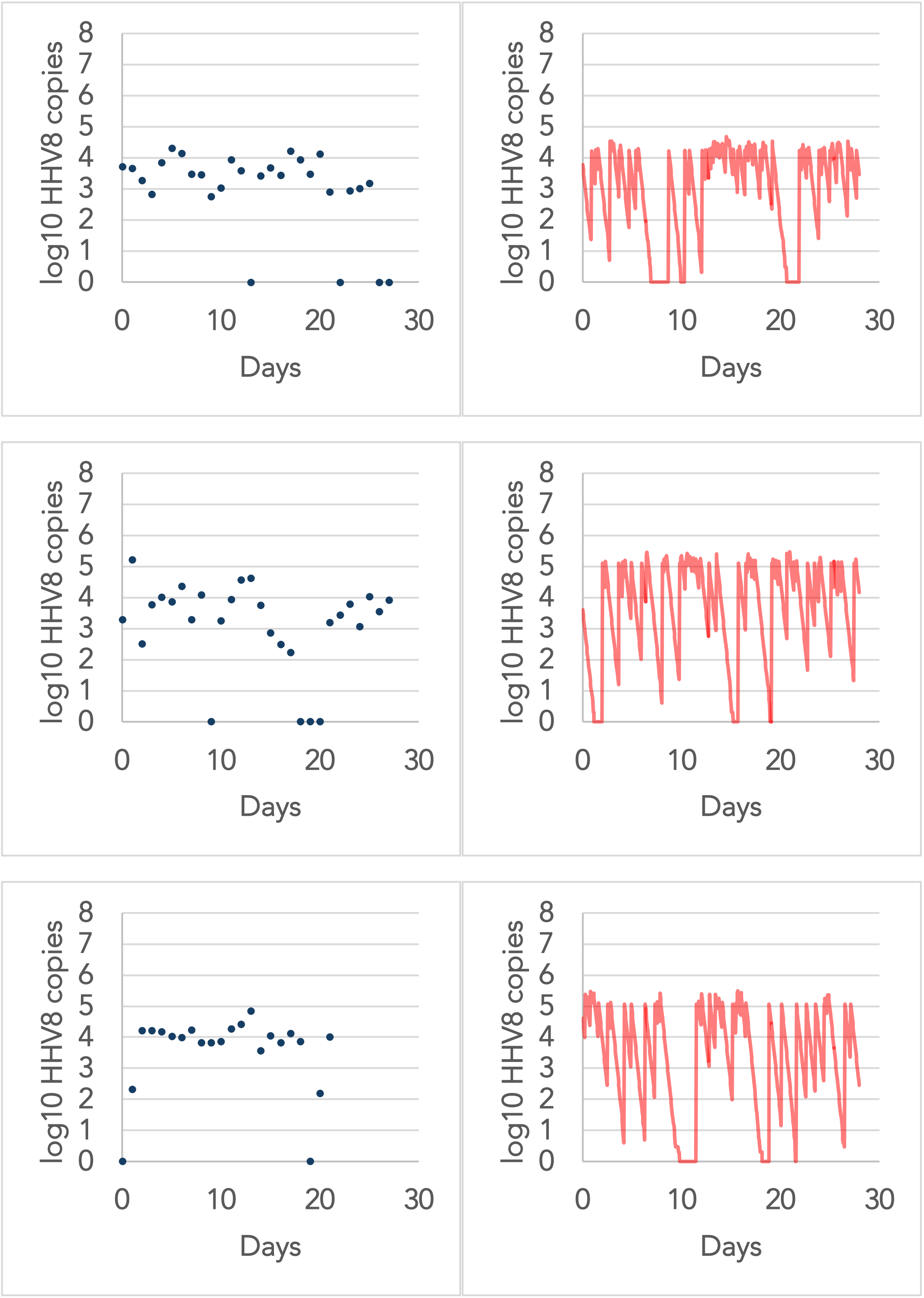
log10 HHV8 copies
0
1
2
3
4
5
6
7
8
0
10
20
30
Days

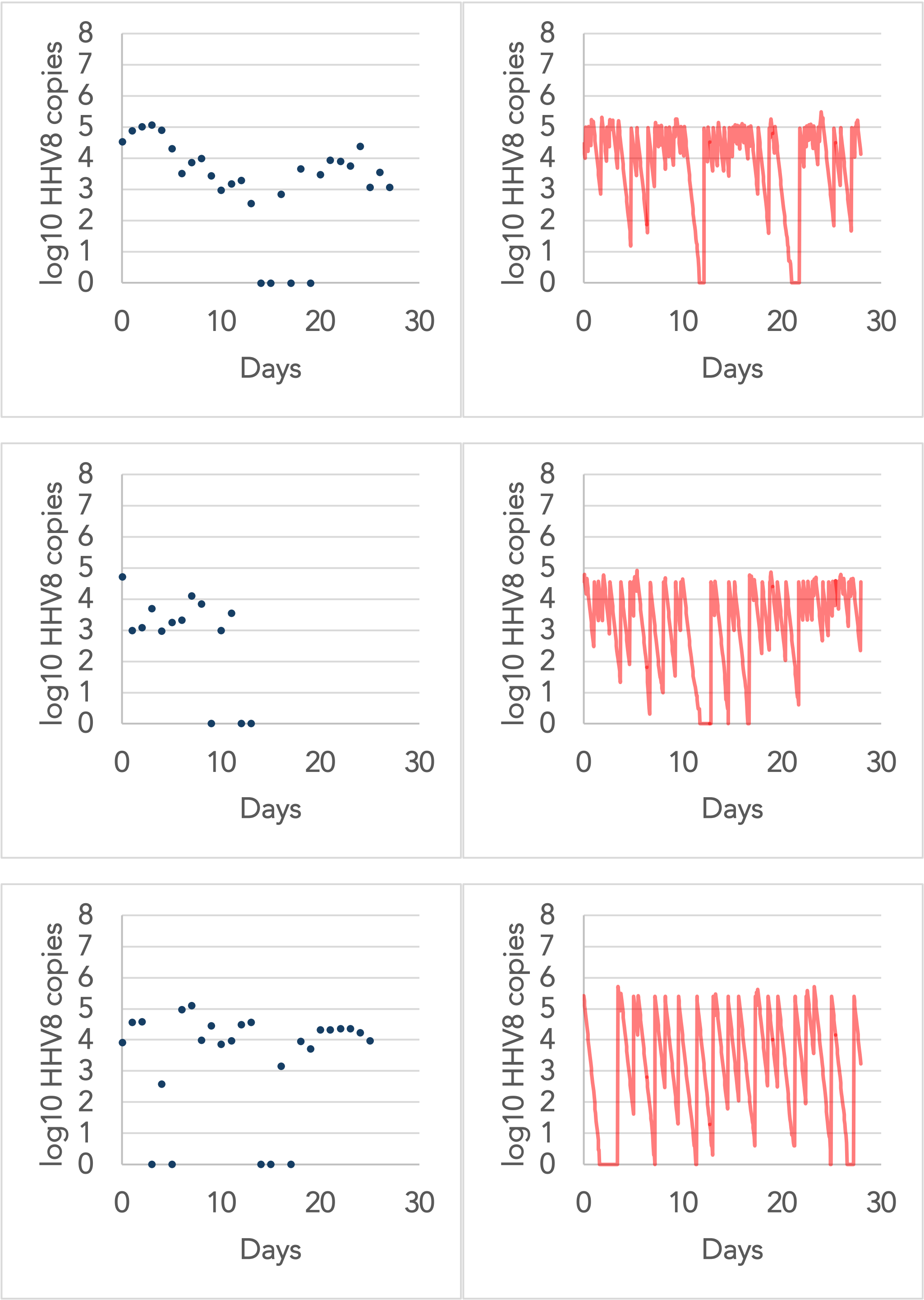

Figure S2. Diverse and accurate viral output from stochastic HHV8 model: examples of frequent epidosic shedding at moderate and fluctuating viral loads from 45 participants. Individual viral loads sampled daily from 10-30 days (blue dots) with stochastic model projections through 30 days (red lines, continuous sampling) using optimal parameter values for each individual. Model output is not intened to reproduce the timing and nature of each HHV8 reactivation but rather overall patterns of shedding. Summary

statistics for model fitting were extracted from daily sampling of model data for fitting to data.

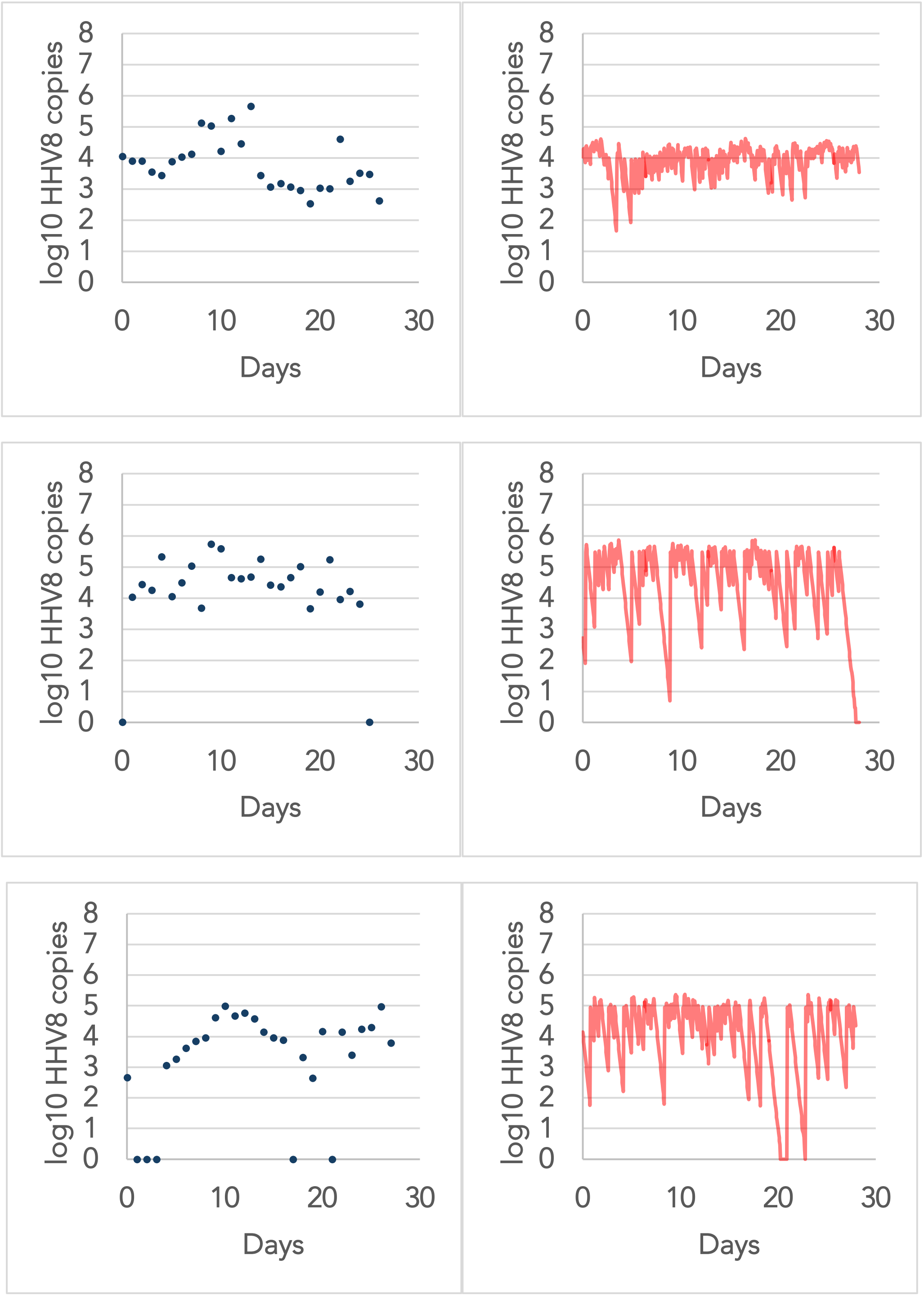
log10 HHV8 copies
0 1 2 3 4 5 6 7 8
0 10 20 30
Days
log10 HHV8 copies
0 1 2 3 4 5 6 7 8
0 10 20 30
Days
log10 HHV8 copies
0 1 2 3 4 5 6 7 8
0 10 20 30
Days
log10 HHV8 copies
0 1 2 3 4 5 6 7 8
0 10 20 30
Days
log10 HHV8 copies
0 1 2 3 4 5 6 7 8
0 10 20 30
Days
log10 HHV8 copies
0 1 2 3 4 5 6 7 8
0 10 20 30
Days

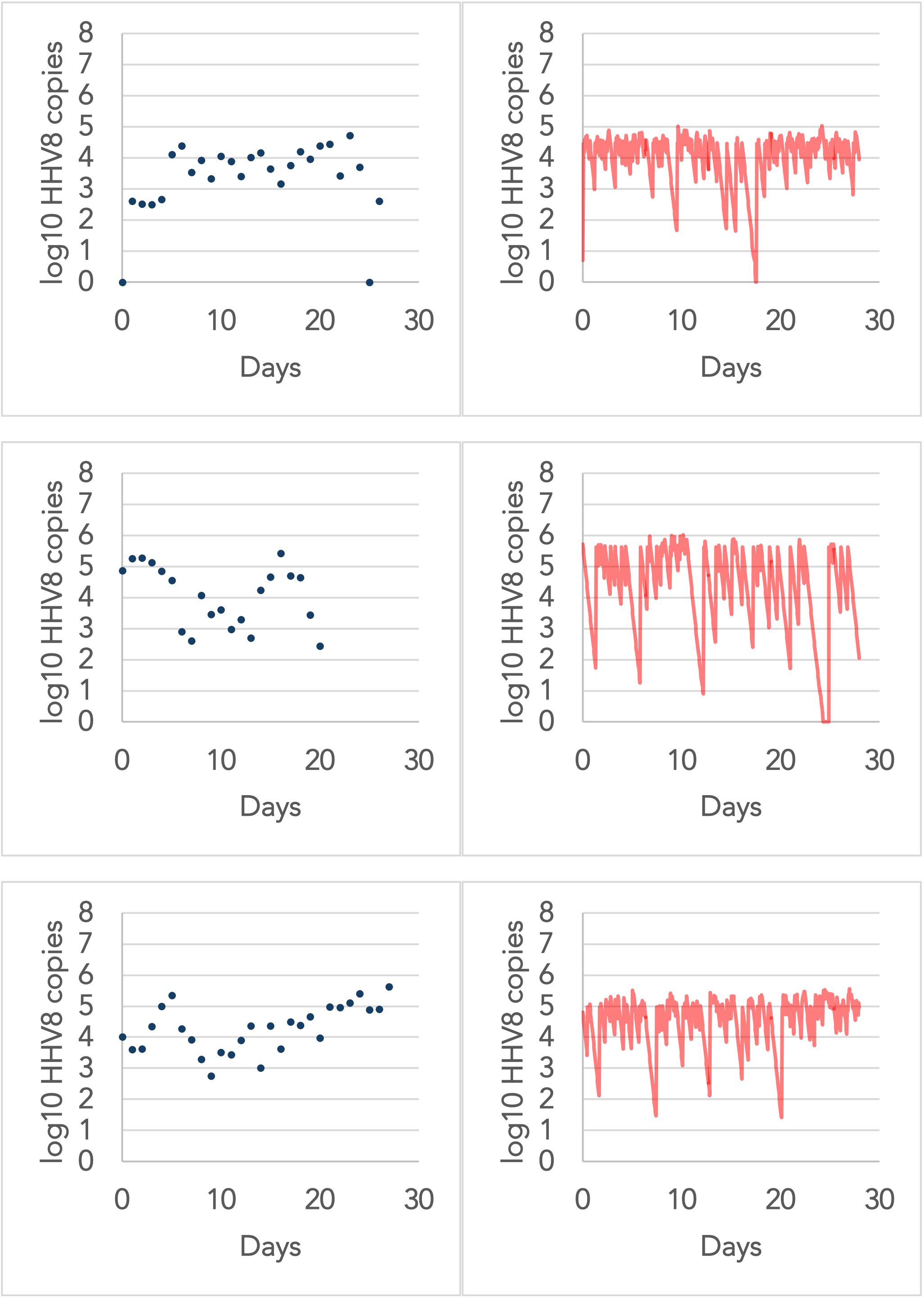
log10 HHV8 copies
Days
log10 HHV8 copies
Days
log10 HHV8 copies
Days
log10 HHV8 copies
Days
log10 HHV8 copies
Days
log10 HHV8 copies
Days

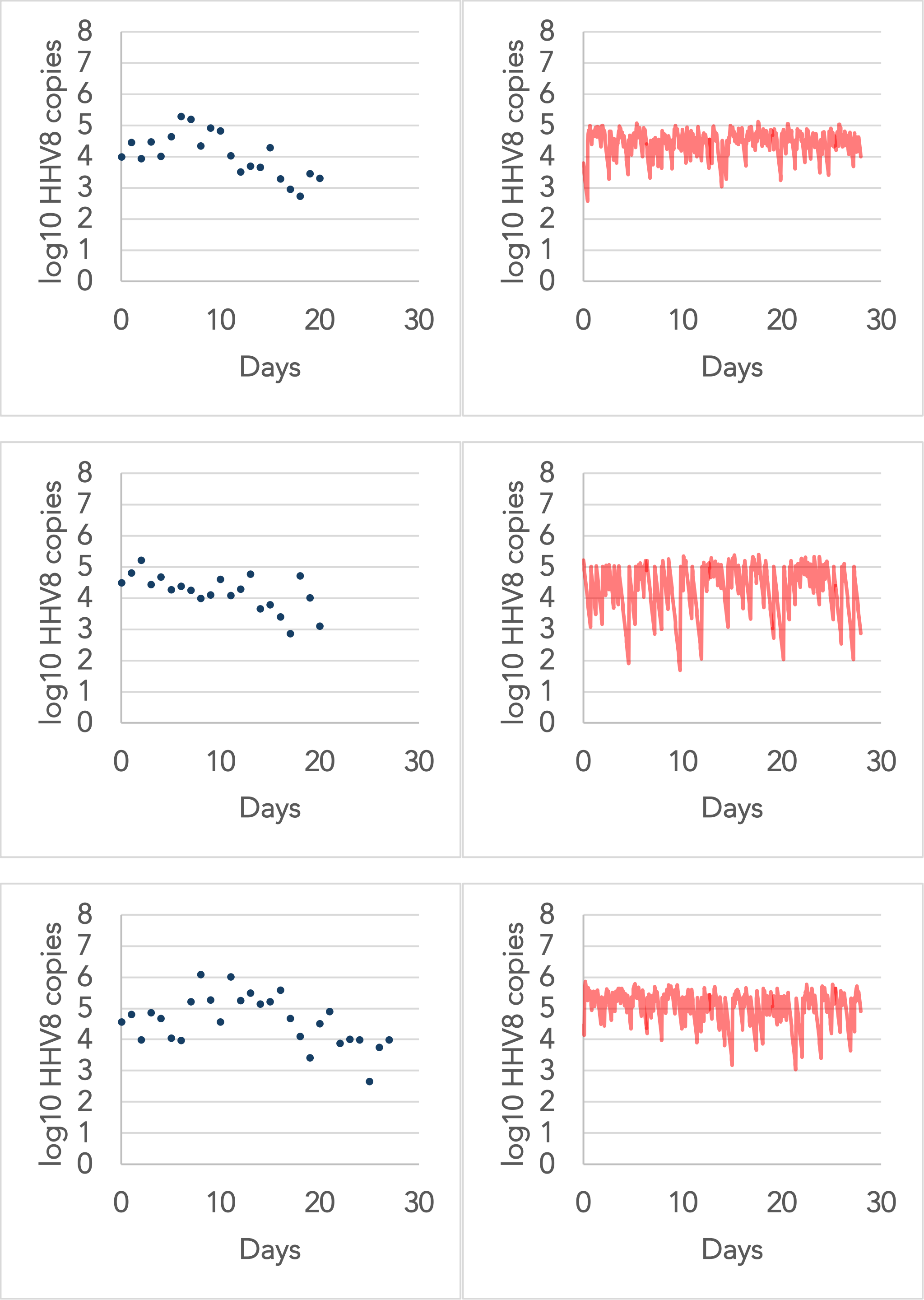

log10 HHV8 copies
0 1 2 3 4 5 6 7 8
0 10 20 30
Days

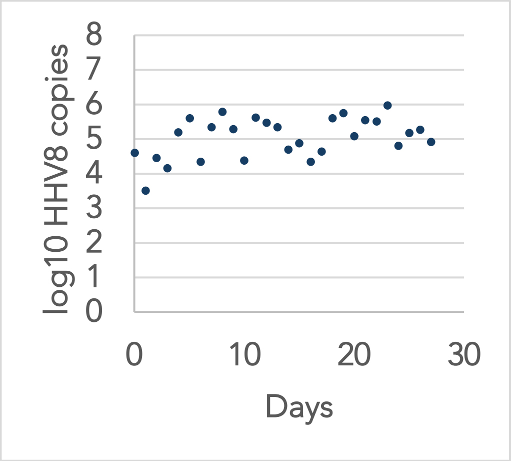
log10 HHV8 copies
Days
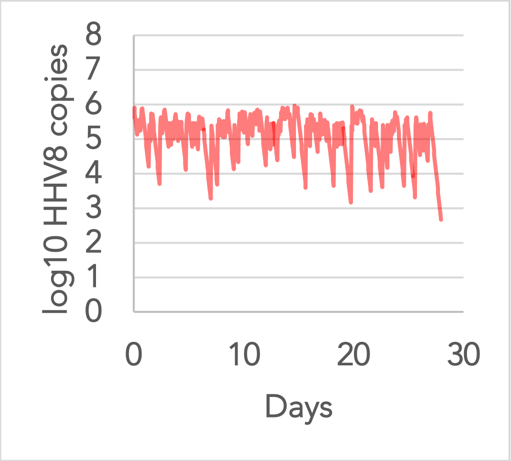
log10 HHV8 copies
Days
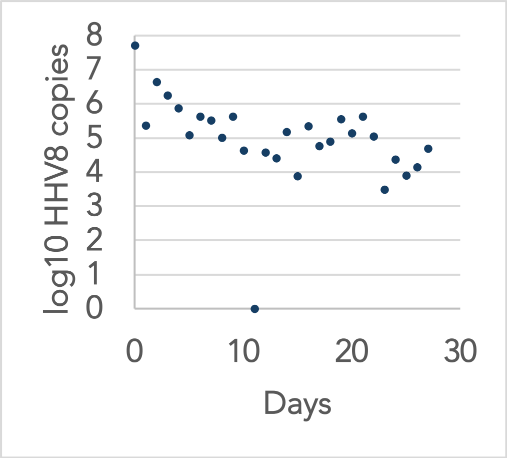
log10 HHV8 copies
Days
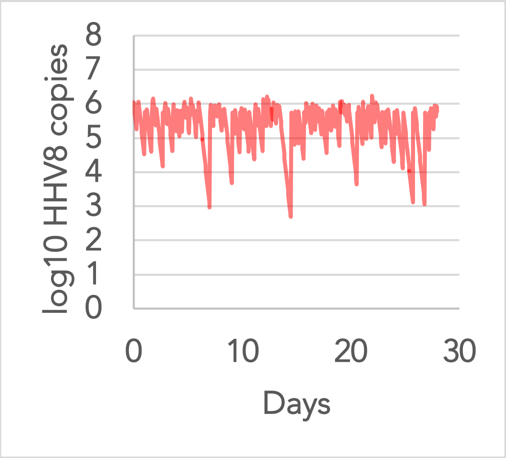
log10 HHV8 copies
Days
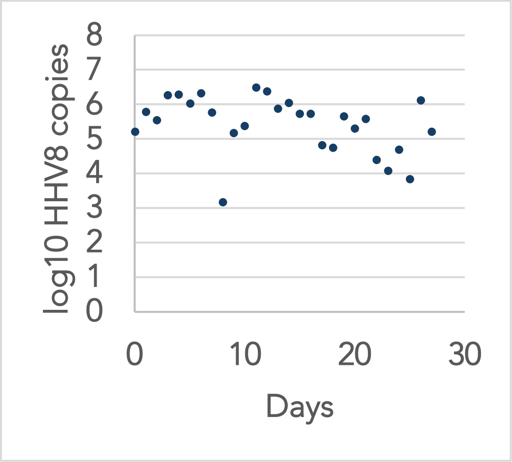
log10 HHV8 copies
Days
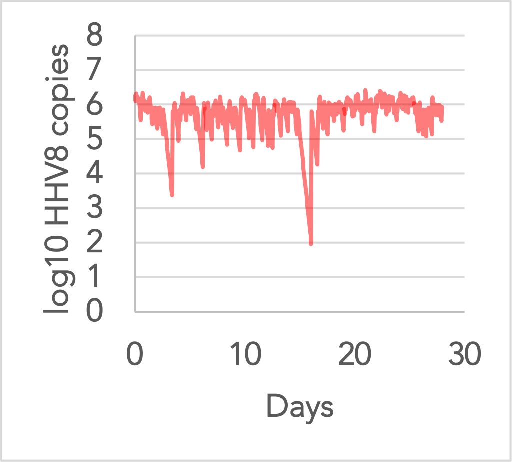
log10 HHV8 copies
Days

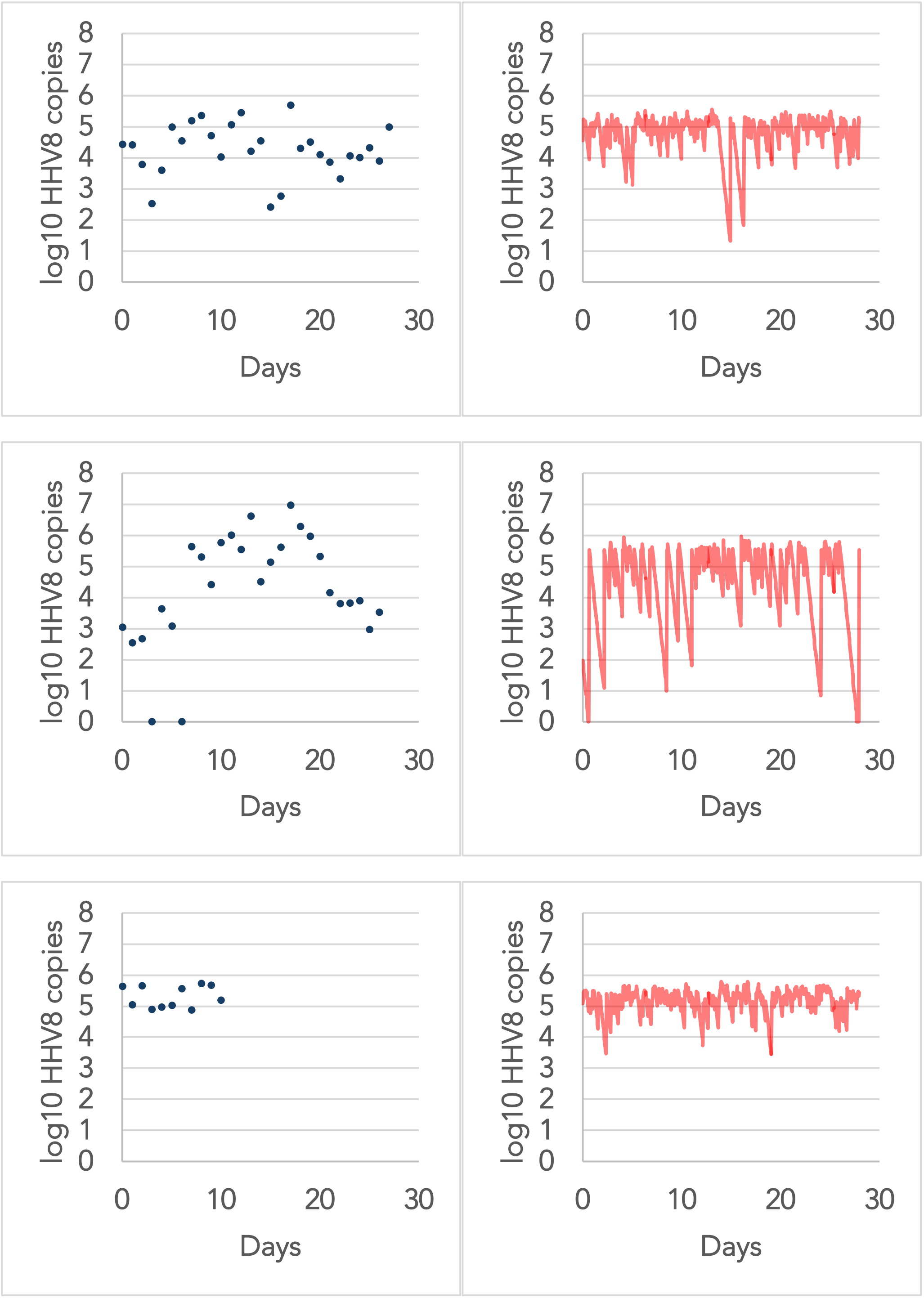
log10 HHV8 copies
Days
0
1
2
3
4
5
6
7
8
10
20
30

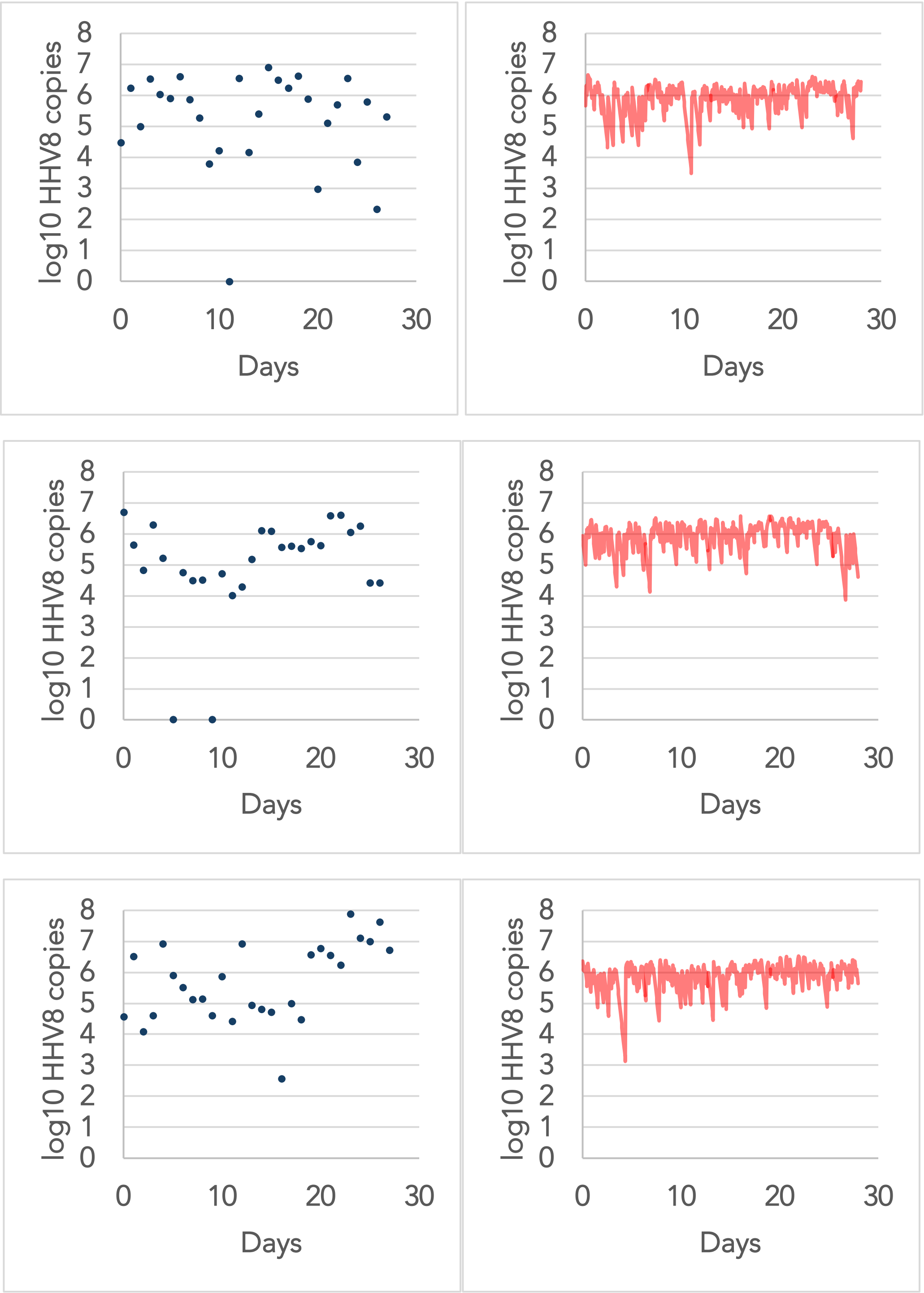
log10 HHV8 copies
Days
0
1
2
3
4
5
6
7
8
0
10
20
30

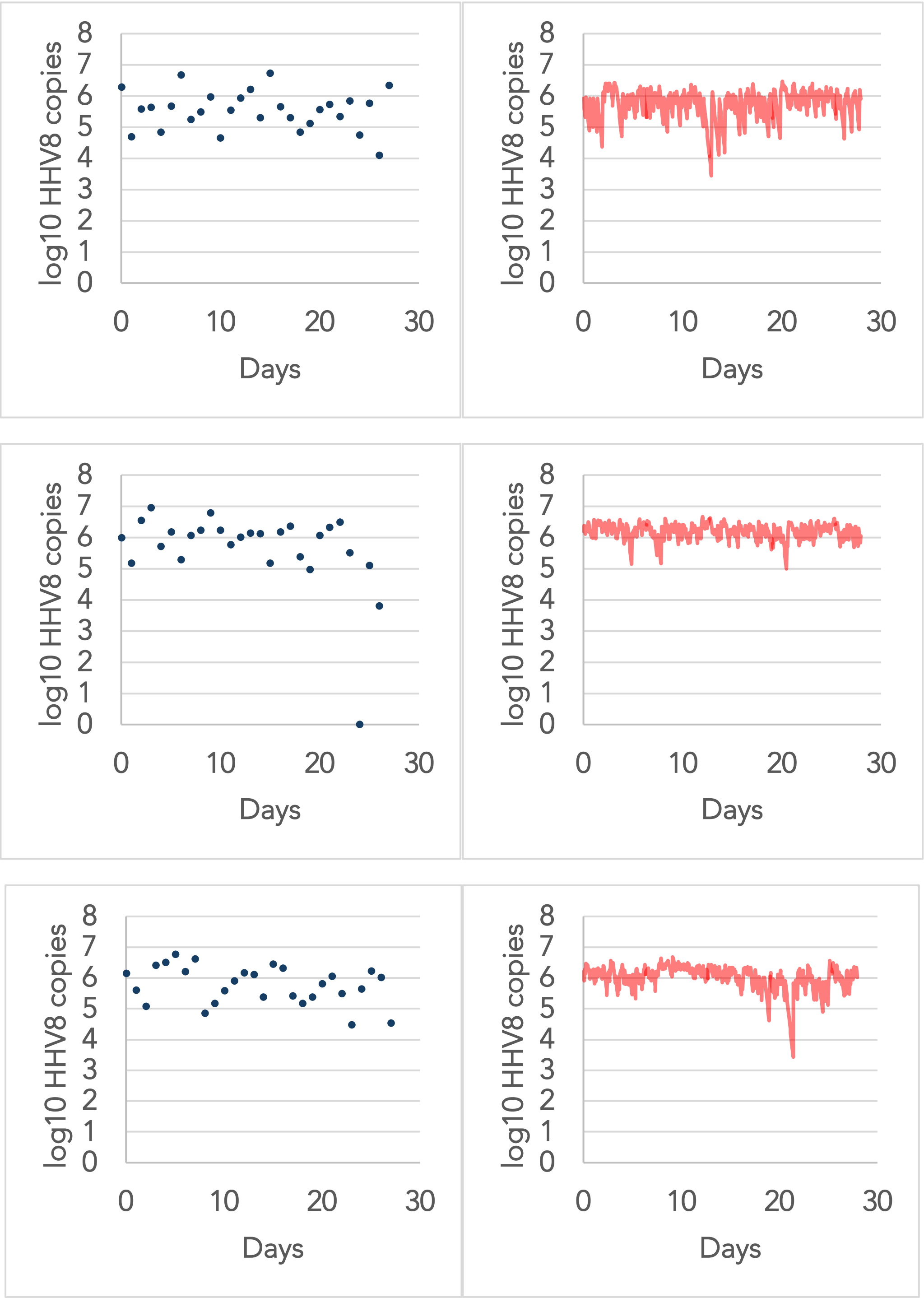
log10 HHV8 copies
0
1
2
3
4
5
6
7
8
0
10
20
30
Days

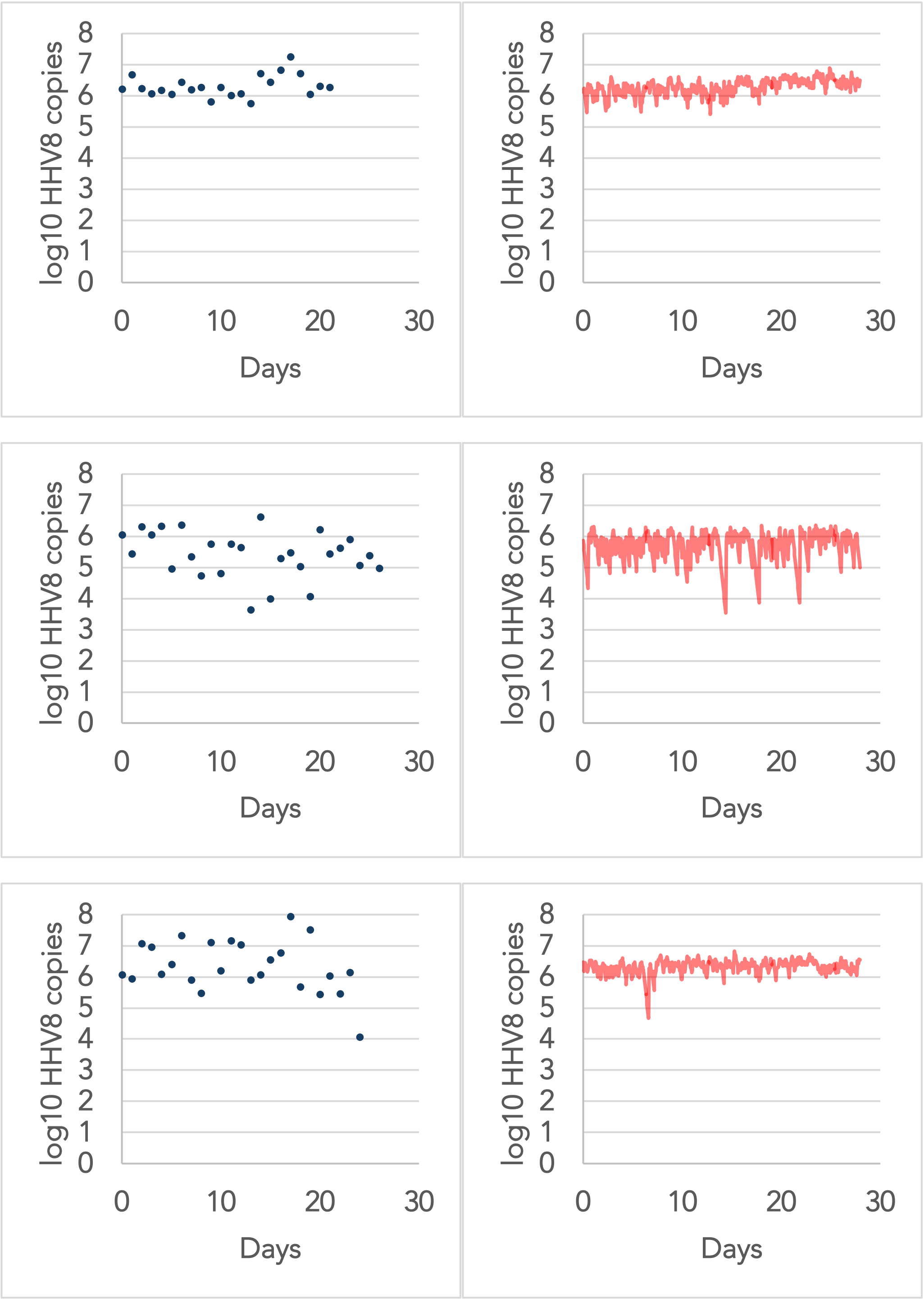

Figure S3. Diverse and accurate viral output from stochastic HHV8 model: examples of persistent or near-persistent shedding at moderate and high viral loads from 24 participants. Individual viral loads sampled daily from 10-30 days (blue dots) with stochastic model projections through 30 days (red lines, continuous sampling) using optimal parameter values for each individual. Model output is not intened to reproduce the timing and nature of each HHV8 reactivation but rather overall patterns of shedding.

Summary statistics for model fitting were extracted from daily sampling of model data for fitting to data.
